# Reconstruction and Dissolution of Copper Catalysts during Electrocatalytic Nitrate Reduction

**DOI:** 10.1021/acs.est.6c04463

**Published:** 2026-06-16

**Authors:** Leslie K. Arrazolo, Kali Rigby, Jorge Moncada, Eli Stavitski, Jae-Hong Kim

**Affiliations:** † Department of Chemical and Environmental Engineering, 5755 Yale University, New Haven, Connecticut 06511, United States; ‡ National Synchrotron Light Source-II, 8099 Brookhaven National Laboratory, Upton, New York 11973, United States

**Keywords:** electrocatalytic nitrate reduction, copper leaching, in situ X-ray absorption spectroscopy, single-atom catalysts

## Abstract

Copper-based electrocatalysts are widely explored for electrochemical nitrate remediation, yet their stability under operating conditions remains poorly understood. While nanoscale and subnanoscale Cu motifs are known to restructure during the nitrate reduction reaction (NO_3_RR), how these transformations translate into irreversible material loss remains unclear. Here, we quantify Cu dissolution during NO_3_RR as a function of catalyst architecture and electrolyte chemistry using two model systems: single-atom Cu (Cu_1_) and Cu nanoparticles (Cu_NP_). Time-resolved leaching measurements, integrated with *in situ* X-ray absorption spectroscopy (XAS), reveal measurable Cu loss for both catalysts during NO_3_RR. Dissolution is concentrated at the initiation of electrolysis, coinciding with rapid restructuring, and depends strongly on morphology, with Cu_NP_ consistently exhibiting greater Cu loss than Cu_1_. *In situ* XAS reveals that Cu_1_ forms transient metallic clusters under reduction that largely redisperse upon returning to open-circuit voltage, whereas Cu_NP_ undergoes reduction of an oxidized surface layer accompanied by sustained Cu loss during electrolysis. Notably, the presence of nitrate significantly intensifies restructuring and Cu loss, highlighting the critical role of electrolyte composition. These findings establish a direct link between electrochemical restructuring and Cu dissolution, unveiling electrolyte-dependent interactions as key determinants of catalyst durability in electrochemical nitrate conversion.

## Graphical abstract

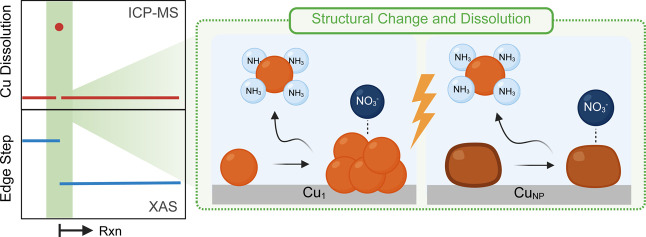

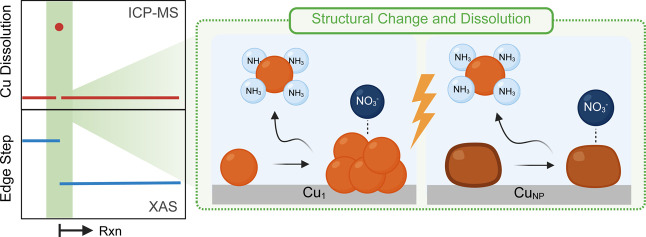

## Introduction

Electrochemical nitrate reduction (NO_3_RR) has emerged as a promising route for ammonia production^1,2^ and wastewater treatment.^3,4^ Among candidate materials, copper-based electrocatalysts have attracted particular attention due to their low cost, high activity, and preferential adsorption of nitrate over protons, which suppresses the competing hydrogen evolution reaction (HER).^5–8^ In particular, Cu catalysts engineered at the nano- and sub-nanoscale have attracted significant interest, as their high surface area and unique electronic structures can enhance reaction rates and selectivity.^9^ Despite these advantages, copper catalysts exhibit significant structural instability under reductive conditions.^10,11^ Catalyst sintering, i.e., particle growth during reaction, presents a major roadblock to catalyst stability. As catalytic materials downsize to the sub-nanoscale, surface free energy dramatically increases, leading to an inherent tendency toward aggregation and restructuring that can be further exacerbated by reaction conditions, ultimately resulting in active site and performance loss.^12,13^ Realizing the promise of Cu catalysts for NO_3_RR therefore requires elucidating active-site dynamics under operating conditions to guide strategies that mitigate instability.

*In situ* and *operando* characterization techniques have been instrumental to probe the chemical state and size evolution of copper nanomaterials during catalytic reactions.^14–19^ *Operando* X-ray absorption spectroscopy (XAS) has shown that Cu single atoms can restructure into metallic Cu nanoparticles concurrent with ammonia (NH_3_) synthesis.^15^ In another work, XAS combined with *operando* transmission electron microscopy (TEM) demonstrated that the rate of Cu_2_O nanocube reduction and restructuring during NO_3_RR is impacted by applied potential and electrolyte composition.^16^ A related study on the electrocatalytic CO_2_ reduction reaction (CO_2_RR) leveraged *in situ* XAS and small-angle X-ray scattering to resolve the structural changes and degradation of Cu oxide nanoparticles, finding that the agglomeration through particle migration and coalescence occurs mainly at the early stages of the reaction.^20^

Despite extensive efforts to unveil the reconstruction mechanism, Cu material loss during the rearrangement of nano- and sub-nanocatalysts under reductive electrochemical conditions remains less explored. Leaching is an important performance metric because it quantifies catalyst material loss that leads to activity loss. In the context of wastewater treatment, dissolved metals are an environmental concern. While metal leaching under oxidation reactions (e.g., oxygen evolution reaction, OER) has been widely studied,^21–24^ our understanding of dissolution under reductive bias is limited and has focused mainly on CO_2_RR. For example, inductively coupled plasma mass spectrometry (ICP–MS) analysis has been used to quantify Cu dissolution from Cu foil during CO_2_RR and HER, showing a strong dependence on applied potential and electrolyte environment, with more alkaline electrolytes generally promoting greater Cu leaching.^25^ In addition, Cu foil dissolution has been also observed during surface oxidation and reduction, with pH dictating the extent of Cu loss.^26^ Factors such as electrolyte composition, applied potential, and catalyst morphology can strongly influence transition-metal dissolution and restructuring during reduction reactions,^20,27–29^ emphasizing the need for systematic studies on Cu catalysts for NO_3_RR.

We here evaluate the leaching and structural transformation of two benchmark Cu catalysts, single atoms (Cu_1_) and nanoparticles (Cu_NP_), during NO_3_RR. Both catalyst morphologies have been reported to undergo significant restructuring at cathodic potentials, making them useful platforms for probing metal dissolution during NO_3_RR.^15,20^ By integrating quantitative leaching measurements with *in situ* XAS, we correlate Cu loss with time-dependent changes in coordination environment for Cu_1_ and Cu_NP_. This work offers insight into how reaction conditions govern the coupled processes of restructuring and leaching in Cu catalysts, providing design considerations for improving catalyst stability during NO_3_RR.

## Experimental Methods

### Materials

All chemicals were of reagent grade or higher and were used as received. Graphene nanoplatelets, nitric acid, hydrochloric acid, Nafion 117, ethanol, copper nitrate, 1,10-phenanthroline, potassium nitrate, potassium sulfate, potassium hydroxide, potassium nitrite, and ammonium chloride were purchased from Sigma-Aldrich. Carbon paper (Toray 120), iridium on carbon, and Aquivion E98-15S proton-exchange membranes were purchased from Fuel Cell Store. Ag/AgCl electrodes were purchased from CH Instruments. Pt foil was purchased from Chemistry Cabinet.

### Catalyst Preparation

The synthesis of Cu_1_ was adapted from Yang et al.^30^ Briefly, 100 mg of 1,10-phenanthroline was dissolved in 40 mL of ethanol, followed by the addition of 200 μL of a 50 mg mL^–1^ Cu­(NO_3_)_2_ solution. After 30 min of mixing, 200 mg of graphene was added. The suspension was shaken overnight at 300 rpm and dried at 80 °C. The resulting powder was ground with a mortar and pestle and pyrolyzed under Ar at 600 °C for 2 h (ramp rate 5 °C min^–1^).

The synthesis of Cu_NP_ was adapted from Wu et al.^31^ Graphene (150 mg) was added to 75 mL of ethanol and sonicated for 1 h. Next, 150 μL of a 50 mg mL^–1^ Cu­(NO_3_)_2_ solution was added and sonicated for additional 30 min. The suspension was then stirred overnight and dried in an oil bath at 70 °C. The resulting powder was ground with a mortar and pestle and pyrolyzed under 5% H_2_/Ar at 500 °C for 3 h (ramp rate 5 °C min^–1^).

Metal loadings were determined by acid digestion using a Milestone Ethos Easy microwave digester. Cu_1_ and Cu_NP_ powders (5 mg) were added to 4.5 mL of HCl and 1.5 mL of HNO_3_ and heated to 230 °C (25 min ramp, 15 min hold). After cooling to room temperature, samples were diluted for ICP–MS analysis (PerkinElmer NexION 5000). The Cu loadings were determined to be 3.8 wt % (Cu_1_) and 4.8 wt % (Cu_NP_), calculated as the mass of Cu per mass of graphene. Catalyst inks were prepared at a ratio of 1 mg of catalyst: 150 μL of ethanol: 3 μL of Nafion. After sonication, 150 μL of ink was drop-cast onto a 0.5 × 1 cm^2^ working area of a carbon paper electrode for batch experiments and 300 μL of ink onto a 0.5 × 2 cm^2^ working area for flow-by experiments. Electrodes were dried under a near-infrared lamp to yield a catalyst loading of 2 mg cm^–2^.

### Characterization

Aberration-corrected high-angle annular dark-field scanning transmission electron microscopy (HAADF-STEM) images and electron energy loss spectroscopy (EELS) spectra were obtained using an aberration corrected Titan Themis AC-STEM (Thermo Fisher Scientific, USA) equipped with a Gatan continuum S (AMETEC, Inc., USA) at the UConn Thermo Fisher Scientific Center for Advanced Microscopy and Materials Analysis at the University of Connecticut. The Themis was operated at 300 keV with a beam current of 0.04 nA for imaging and 0.4 nA for EELS. Imaging was done using 130 mm camera length and a HAADF detector. The EELS convergence angle and collection angle are 25 and 50 mrad, respectively. 0.3 eV/pixel is used to simultaneously cover O K-edge (532 eV) and Cu L-edge (931 eV) for their elemental distribution. Surface powders from Cu_1_ and Cu_NP_ electrodes were gently scraped with a spatula and transferred onto TEM grids. Post-reaction imaging was performed on electrodes subject to reduction for 8 h at −0.8 V in 0.1 M KNO_3_ + 0.1 M K_2_SO_4_ at pH 11.5.

XAS was performed at Beamline 8-ID of the National Synchrotron Light Source II at Brookhaven National Laboratory. *Ex situ* samples were measured in transmission mode. Raw data were processed using Athena software to generate μ­(E) spectra, including background subtraction and normalization, and to perform Fourier transformation and plotting. Spectra were calibrated using Cu foil references. Extended X-ray absorption fine structure (EXAFS) fitting was carried out using Artemis software, with reference samples used to determine S_0_^2^ parameters prior to fitting.

*In situ* XAS experiments were performed in a custom, undivided cell with a window that held the working electrode. The working electrode consisted of a 1 × 1 cm^2^ coated area of Cu_1_ or Cu_NP_ at 2 mg cm^–2^. The electrolyte was 0.1 M KNO_3_ + 0.1 M K_2_SO_4_ or 0.15 M K_2_SO_4_, with pH adjusted to 11.5 using KOH, and was bubbled with He at 3 sccm. An Ag/AgCl electrode was used as the reference and a sheet of carbon paper as the counter electrode. Fluorescence mode data were collected using a four-element silicon drift diode detector coupled to an Xspress3 readout module.^32^ Continuous scans were collected for 2 h (∼1 scan per minute).

### Nitrate Reduction Experiments

Nitrate reduction was performed in a divided H-cell separated by a proton exchange membrane. The electrochemical surface areas of the catalysts and the support were determined using a glassy carbon electrode (Figures S1–S5). Pt foil and Ag/AgCl were used as the counter and reference electrodes, respectively. In this study, all potentials were converted to the reversible hydrogen electrode, and voltages were not *iR*-corrected. The electrolyte (10 mL catholyte, 5 mL anolyte) consisted of 0.1 M KNO_3_ + 0.1 M K_2_SO_4_, 0.05 M KNO_3_ + 0.12 M K_2_SO_4_, or 0.15 M K_2_SO_4_, with pH adjusted to 11.5 by KOH. Electrodes were placed in the catholyte for 30 min under Ar purging to allow equilibration, after which a 200 μL aliquot was collected. Electrochemical reactions were conducted for 1, 3, 5, or 8 h at applied potentials of −0.4, −0.6, and −0.8 V. Additional 200 μL catholyte aliquots were collected at the conclusion of the reaction while the potential was still applied. After returning to open circuit voltage (OCV), electrodes were left in the catholyte for 5 min before taking final samples. Each time point, Cu_t_, was obtained from an individual reactor. Cu leaching was calculated as described in Text S1.

For flow-by experiments, iridium on carbon was deposited at a loading of 1 mg cm^–2^ for the anode. Both electrodes (geometric area = 1 cm^2^) were placed on opposite sides of a polytetrafluoroethylene (PTFE) chamber housing a 0.5 cm × 2 cm flow channel for the electrolyte. The cathode was fed with the indicated electrolyte at 0.1 mL min^–1^ and was Ar-saturated, whereas the anode was recirculated with 0.1 M K_2_SO_4_ (pH = 6) at 1 mL min^–1^ using a peristaltic pump (VMR). An Aquivion E98-15S membrane was placed adjacent to the anode. The interelectrode spacing was 5 mm. The cell was fastened using a titanium current collector on the anode and a stainless-steel current collector on the cathode. Flow-by evaluations were performed at constant current to simulate kinetically controlled conditions and at a relatively low current density of –20 mA cm^–2^ (geometric area basis) to minimize leaching associated with enhanced mixing from H_2_ evolution.^25^ A sample aliquot (0.8 mL) of cell effluent was collected at the indicated time point, while the reaction proceeded and Cu leaching was calculated as outlined in Text S2. Nitrate, nitrite, and ammonium concentrations were measured after acidifying samples with H_2_SO_4_ using ion chromatography (Metrohm 940 Professional IC Vario). ICP–MS samples of leached Cu were diluted in 1% HNO_3_. Each data point represents the mean of three independent measurements (*n* = 3) with error bars representing the standard deviation of the data.

## Results and Discussion

### Catalyst Morphology Determines Extent of Leaching

We first evaluated the influence of morphology on Cu catalyst stability during NO_3_RR using two benchmark catalysts: Cu_1_ and Cu_NP_. HAADF-STEM confirmed the presence of atomically dispersed Cu_1_, as well as Cu_NP_ with an average diameter of ∼20 nm (Figure 1a,b). X-ray absorption near-edge structure (XANES) spectra further supported the single-atom configuration of Cu_1_, while Cu_NP_ appeared more oxidized than zero-valent Cu foil (Figure 1c). This is consistent with a metallic Cu core and an oxidized copper shell observed for Cu_NP_ by EELS analysis (Figure S6), which is likely formed due to air exposure or during ink preparation.^17^ In the EXAFS spectra, the main peak of Cu_1_ aligned with the Cu–N coordination observed in copper phthalocyanine (CuPc) (Figures 1d and S7). Coordination number calculations indicated Cu–N_4_ coordination, with a Cu–N coordination number (CN) of 4.10 ± 0.25 (Table S1). In contrast, Cu_NP_ exhibited a dominant peak at ∼2.3 Å corresponding with Cu–Cu binding in metallic Cu (Cu foil), along with a weaker peak at ∼1.6 Å for Cu–O coordination, supporting a metallic Cu nanoparticle structure with an oxidized surface layer (Figures 1d and S8).

**Figure 1:**
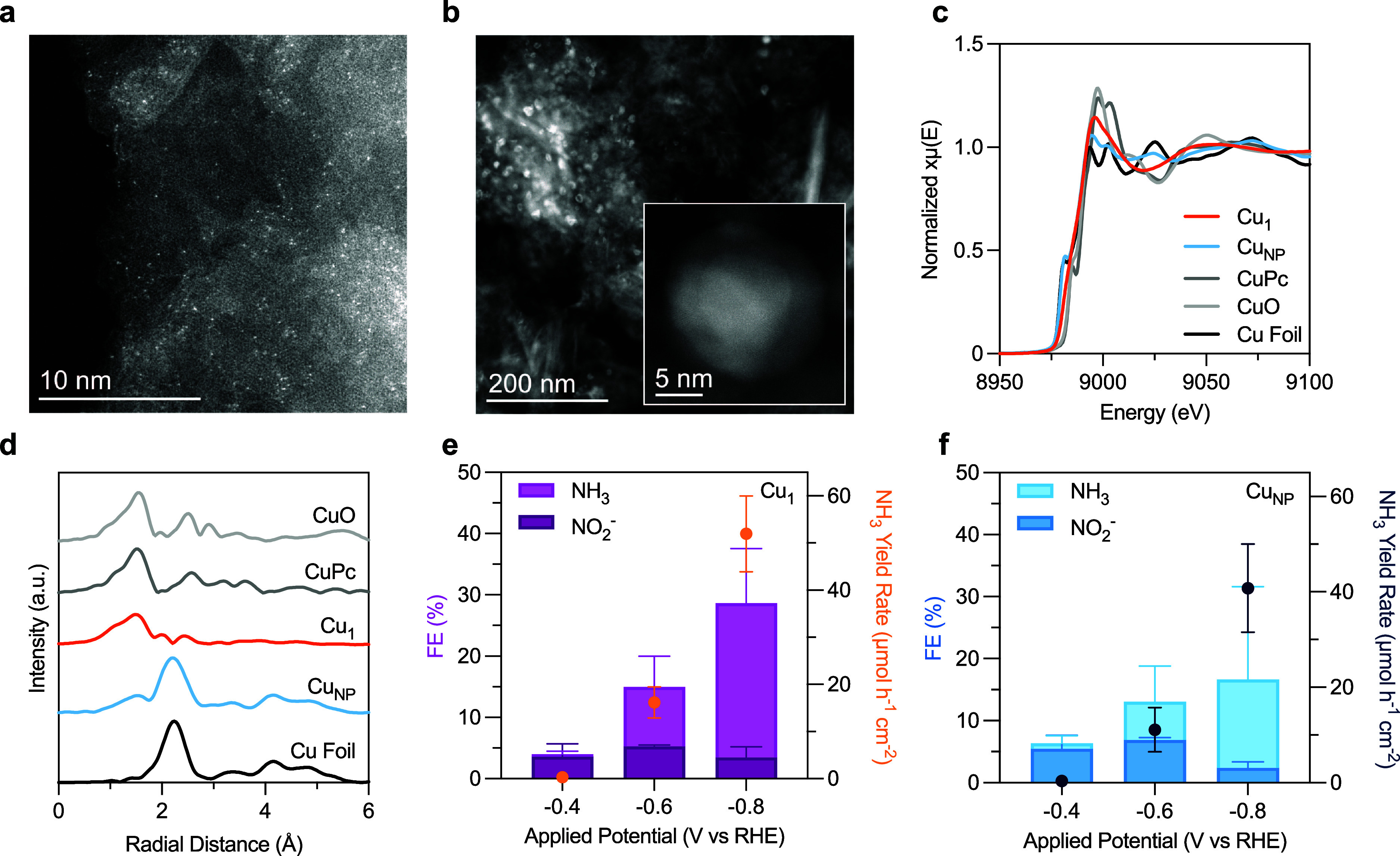
Characterization and catalytic performance of Cu_1_ and Cu_NP_. HAADF-STEM images of (a) Cu_1_ and (b) Cu_NP_; (c) XANES and (d) EXAFS spectra of Cu catalysts and references (Cu_1_ × 1.5 and Cu foil × 0.5). Nitrate conversion performance (Faradaic efficiency (FE) and ammonia yield rate) of (e) Cu_1_ and (f) Cu_NP_ at various applied potentials after 8 h of electrolysis.

We conducted batch nitrate reduction reactions for 8 h at −0.4, −0.6, and −0.8 V to evaluate the NO_3_RR performances of Cu_1_ and Cu_NP_ in 0.1 M KNO_3_ + 0.1 M K_2_SO_4_ to minimize HER contributions. Increasing the applied reductive potential to −0.8 V increased both the total nitrate conversion and FE for NH_3_ over NO_2_^–^ (Figure 1e,f). From linear sweep voltammetry, an applied potential of −0.8 V corresponds to current densities −45 and −98 mA cm^–2^ for Cu_1_ and Cu_NP_, respectively (Figure S9). At −0.8 V, nitrate removal increased with reaction time, while FE toward NH_3_ decreased for both catalysts (Figure S10). After 8 h, nitrate removal reached ∼60% for both Cu_1_ and Cu_NP_, with Cu_1_ exhibiting a higher FE for NH_3_ than Cu_NP_. This trend is consistent with previous reports showing that as-synthesized Fe and Cu single atoms outperform nanoparticle counterparts for nitrate reduction, which has been attributed to lower thermodynamic barriers at isolated sites and more favorable adsorption of the reactants.^33,34^

We also monitored Cu leaching before, during, and after the NO_3_RR reactions to gain insights into dissolution behavior differences between Cu_1_ and Cu_NP_ across reaction times and potentials. Figure 2a summarizes the sampling protocol for an experiment performed over duration *t*: Cu_pre_, Cu_t_, and Cu_post_. For each experiment, Cu_pre_ was collected after 30 min at OCV to measure Cu leaching from loosely bound catalyst detaching from the surface. The catholyte was then sampled after *t* = 1, 3, 5, or 8 h of operation to obtain Cu_t_, which captures the extent of Cu leaching under an applied cathodic potential. Each Cu_t_ value was obtained from an independent experiment at the corresponding duration and therefore does not represent time-series data. After the completion of electrolysis, the cell returned to OCV for 5 min to allow desorption of unstable Cu species, after which the final sample, Cu_post_, was collected.

**Figure 2:**
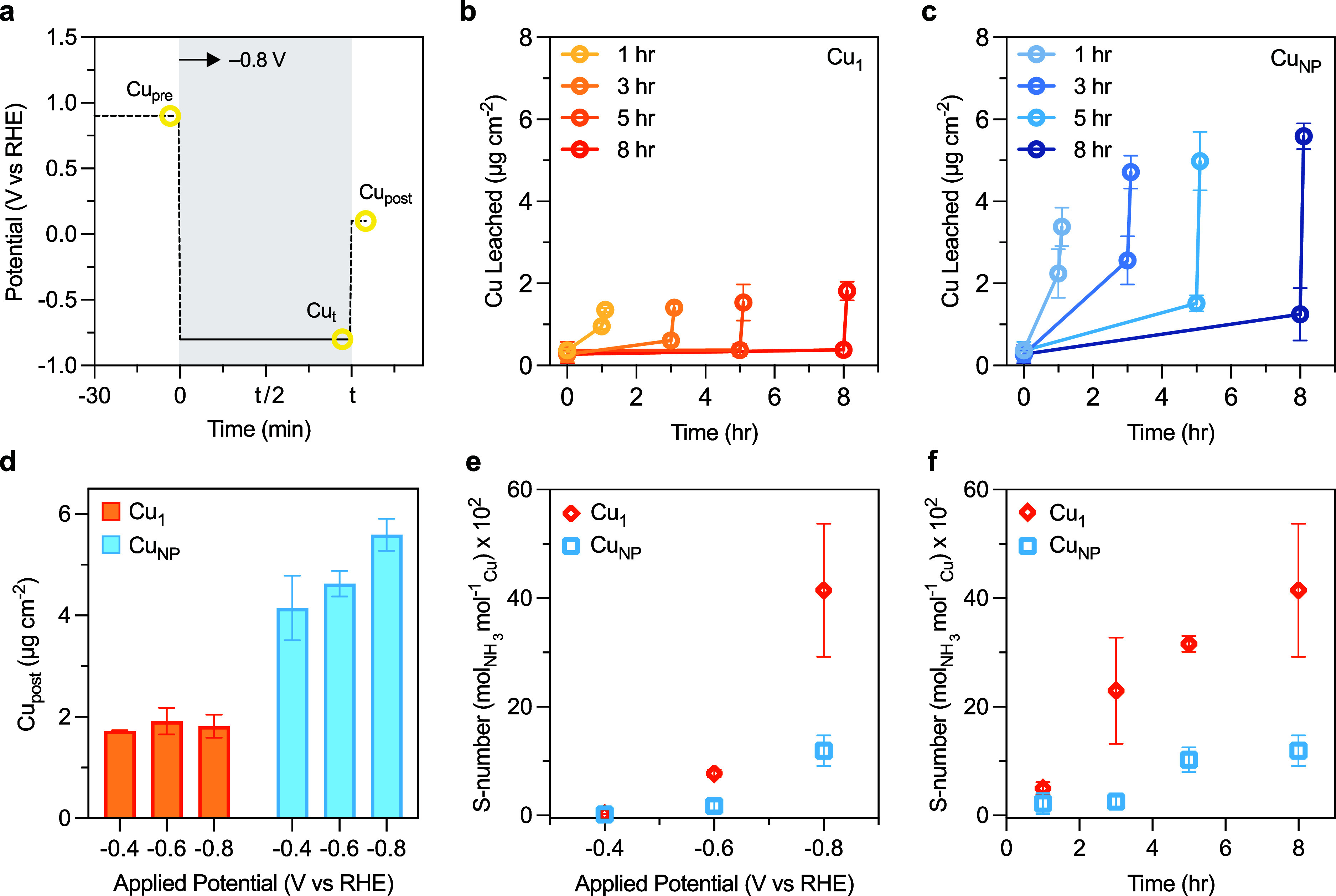
Copper leaching at various reaction times and potentials for Cu_1_ and Cu_NP_ in a batch configuration. (a) NO_3_RR reaction voltage profile with yellow circles indicating sampling events. Copper leaching profiles for (b) Cu_1_ and (c) Cu_NP_ at −0.8 V for 1, 3, 5, and 8 h of NO_3_RR. Samples were taken after 30 min at OCV, at the reaction time point, and 5 min post reaction at OCV. (d) Post-reaction leaching as a function of applied potential. S-numbers for Cu_1_ and Cu_NP_ by (e) varied applied potentials for 8 h and (f) varied reaction times at −0.8 V.

Initial Cu leaching was minimal for both Cu_1_ and Cu_NP_, generally under 0.63 μg cm^–2^ (∼0.5% total Cu loss). As the NO_3_RR duration increased, differences between Cu_1_ and Cu_NP_ emerged. Cu_1_ consistently leached less than Cu_NP_ under all conditions, while both catalysts exhibited the same overall trend: Cu_t_ decreased and Cu_post_ increased with increasing NO_3_RR duration. At −0.8 V after 8 h, Cu_post_ was 1.81 μg cm^–2^ for Cu_1_ and 5.59 μg cm^–2^ for Cu_NP_ (Figure 2b,c). In addition, Cu_1_ leaching showed only a weak dependence on potential, whereas Cu_post_ for Cu_NP_ increased substantially at more reductive potentials (Figures 2d and S11 and Table S2), consistent with the reduction of oxidized Cu species at the nanoparticle surface.^16^

To further analyze the effect of reaction conditions on Cu catalyst stability, we calculated a stability number (S-number),^22^ defined here as the mole ratio of NH_3_ produced to the total amount of copper leached after the completion of each NO_3_RR experiment (i.e., after returning to OCV, Cu_post_). The S-number therefore quantifies resistance to Cu leaching during reaction: a higher S-number indicates less Cu loss for a given extent of NH_3_ production (Text S3). For experiments conducted for 1–8 h of NO_3_RR at −0.4 V, the S-number showed minimal variability due to low NH_3_ production under these potentials (Figures 2e and S12a). For experiments at −0.6 V, a slight volcano trend appears likely due to trade-off between ammonia production and Cu leaching, with intermediate reaction times yielding the highest ammonia production per Cu lost (Figure S12b). At −0.8 V (i.e., a more reductive potential), the S-number increased with increasing reaction duration (Figure 2f). Although more Cu leached with longer NO_3_RR duration for both catalysts, the S-number did not remain constant, indicating that Cu loss did not scale linearly with the loss of catalytic efficiency. Instead, stability increased with reaction time, with a more pronounced increase for Cu_1_ than for Cu_NP_, suggesting that Cu_1_ is less susceptible to NO_3_RR-induced leaching. For Cu_NP_, the greater loss of Cu associated with reduction and dissolution of oxidized surface species likely contributed to its lower stability relative to Cu_1_.^16^

Prior studies of Cu catalysts for CO_2_RR and NO_3_RR have attributed Cu nanoparticle dissolution in part to high-pH environments. Although Cu(s) is the only thermodynamically stable species under the bulk solution pH and applied potential, *operando* Raman studies have reported the coexistence of metallic and oxidic phases over extended operation, consistent with hydroxide formation as a possible dissolution pathway.^16,26^ The limited increase in the Cu_NP_ S-number with time is therefore likely associated with an increase in local pH, which can promote Cu­(OH)_2_ formation and subsequent dissolution. In contrast, Cu_1_ might not follow this pathway due to different Cu–substrate interactions.

From batch reactions, we observed measurable Cu accumulation in the bulk electrolyte due to the reductive applied potential. In addition, Cu leaching was consistently greater for Cu_NP_ than for Cu_1_ and depended on reaction time and applied potentials, whereas Cu_1_ leaching showed only a weak dependence. Cu lost during electrolysis likely accumulated near the electrode surface during NO_3_RR due to electrostatic interactions and subsequently redistributed into the bulk solution once the cathodic bias was removed (i.e., OCV after completion of the batch experiment). To better understand the source of this leaching, the operation in flow-by mode was evaluated.

### Cu Catalyst Leaching at the Onset of NO_3_RR

Next, we conducted NO_3_RR in a flow-by reactor under constant current, monitoring both nitrate removal and Cu dissolution. Both nitrate removal and FE toward NH_3_ remained stable across the 8 h reaction (Figure S13). Prior to electrolysis, Cu_1_ and Cu_NP_ electrodes were equilibrated at OCV under electrolyte flow for 1 h to ensure measured Cu originated primarily from electrolysis rather than mechanical detachment induced by flow shear. ICP–MS analysis showed a pronounced Cu spike at the first sampling point for both catalysts during the OCV period. We attribute this initial Cu leaching to contact dissolution and/or loss of loosely bound catalyst upon initial exposure to the flowing electrolyte (Figure S14).^35^ Note that this point was excluded from Figure 3 to emphasize leaching associated with NO_3_RR. Flow-by experiments reveal that the dominant leaching event occurred at the start of electrolysis, during the transition from OCV to applied current (Figure 3a), with minimal leaching during the remainder of the 8 h experiment. From these steady-state dissolution rates (during 1–8 h), we might expect that any continued dissolution at longer time scales would proceed at a similar rate. Consistent with trends observed in batch experiments, leaching at the reaction onset was greater for Cu_NP_ than for Cu_1_.

**Figure 3:**
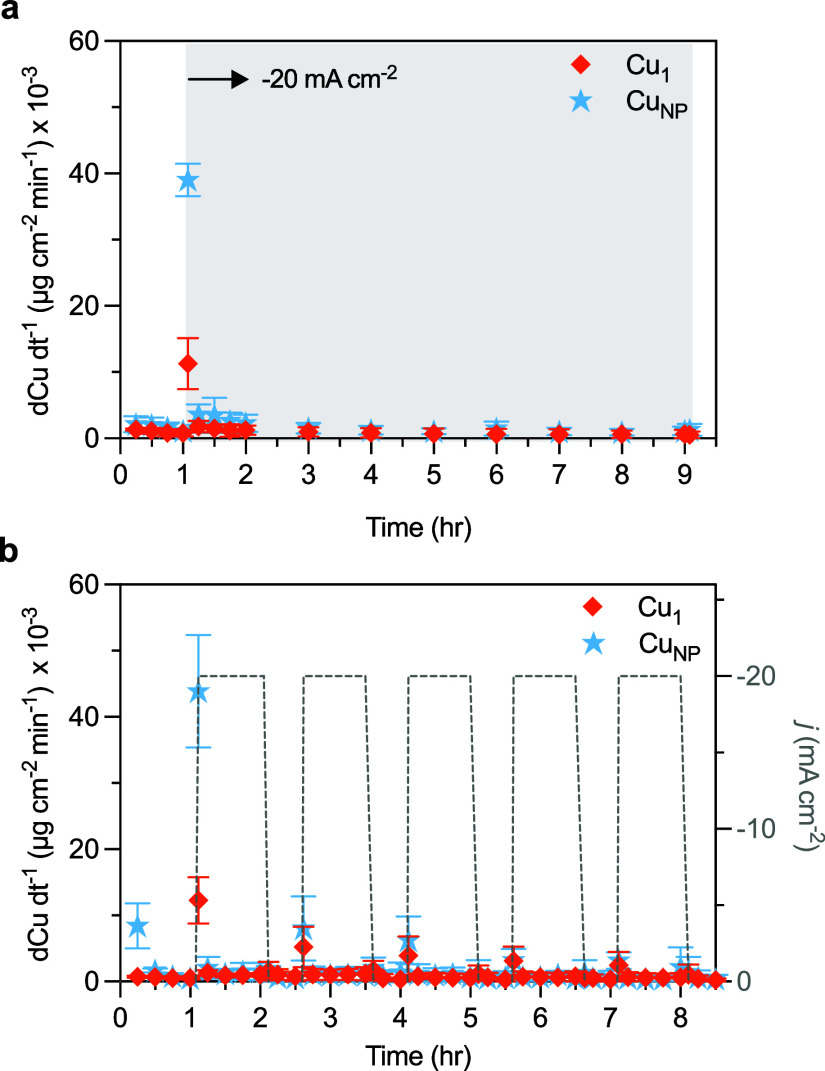
Copper leaching time profiles measured in a flow-by configuration. (a) Cu leaching for Cu_1_ and Cu_NP_ during a 1 h equilibration period at OCV, followed by galvanostatic operation at −20 mA cm^–2^ for 8 h in 0.1 M KNO_3_ + 0.1 M K_2_SO_4_, pH 11.5. Samples were collected every 15 min during equilibration and first hour of electrolysis and then hourly for the remainder of the experiment; a final sample was taken at OCV post reaction. (b) Cu leaching from Cu_1_ and Cu_NP_ under “on/off” pulsed operation, with an initial 1 h equilibration at OCV and 5 cycles consisting of 1 h at −20 mA cm^2^ (“on”) followed by 30 min at OCV (“off”).

Pulsing is a common strategy to reverse or mitigate catalyst transformations during CO_2_RR and to enhance reactant mass transport during NO_3_RR.^2,36,37^ In addition, electrocatalytic systems in a practical setting may operate only intermittently.^38^ Although prior reports of pulsed electrolysis for NO_3_RR and CO_2_RR have noted Cu and Fe leaching,^39,40^ leaching during pulsed NO_3_RR has not been systematically quantified. We therefore implemented a pulsing pattern to simulate repeated “on/off” operation. In these experiments, leaching at the current onset was greater for Cu_NP_ than for Cu_1_, whereas leaching during steady operation was comparable between the two catalysts (Figure 3b). For both catalysts, the magnitude of leaching decreased as the number of pulses increased, and leaching during the “off” periods was minimal (all values fell below ∼2 μg cm^–2^ min^–1^). Despite the onset leaching in each cycle, Cu loss did not measurably affect nitrate removal or FE for either catalyst under this cycling procedure (Figure S15), with total Cu losses of 2.4% and 2.6% of the total Cu loading for Cu_1_ and Cu_NP_, respectively (Table S3).

### Nitrate Promotes Cu Leaching

We next investigated the competing HER and relative roles of nitrate on Cu stability during NO_3_RR. We conducted batch leaching experiments with varying nitrate concentrations but similar conductivity (Table S4) following the sampling procedure in Figure 2a. In the nitrate-free electrolyte (0.15 M K_2_SO_4_, pH 11.5), HER is the only cathodic reaction. After 8 h at −0.8 V under HER-only conditions, leaching was negligible at Cu_pre_ and Cu_t_ (Figure S16). After returning to OCV, measured Cu increased, indicating that HER also induced Cu leaching for both Cu_1_ and Cu_NP_. Notably, Cu_post_ was approximately three times higher for Cu_NP_ than for Cu_1_, demonstrating that Cu_NP_ was more susceptible to leaching in HER-only conditions. Examining Cu leaching as a function of nitrate concentration (0, 0.05, and 0.1 M), we observed that Cu_post_ increased with increasing nitrate concentration for both catalysts (Figure 4a,b). Relative to the nitrate-free condition, Cu_post_ increased by 184% for Cu_1_ and 77% for Cu_NP_. Although differences in anion composition (e.g., SO_4_^2–^ versus NO_3_^–^/SO_4_^–^) may lead to higher interfacial K^+^ concentration and HER activity, these effects do not account for the observed trends. Instead, the presence of nitrate promotes Cu leaching for both catalysts, exerting a stronger relative influence on Cu_1_ despite greater absolute Cu loss from Cu_NP_.

**Figure 4:**
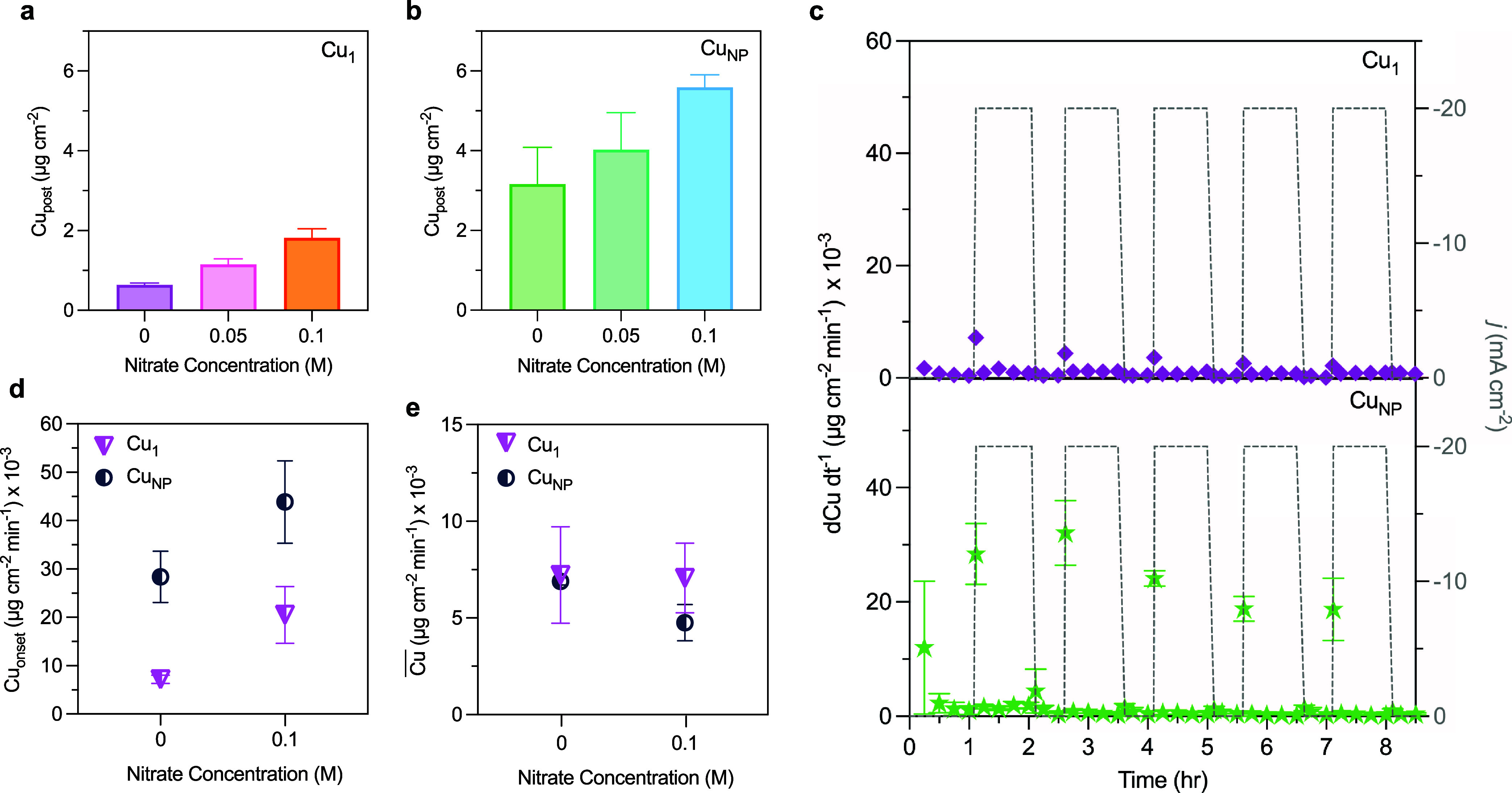
Copper leaching as a function of nitrate concentration. Copper leaching in electrolytes containing no added nitrate (0.15 M K_2_SO_4_, pH 11.5) and halved nitrate concentration (0.05 M KNO_3_, pH 11.5), compared to 0.1 M KNO_3_ condition, during 8 h of batch electrolysis at −0.8 V for (a) Cu_1_ and (b) Cu_NP_. (c) Copper leaching under on–off pulsed operation for (upper) Cu_1_ and (lower) Cu_NP_ with an initial 1 h equilibration at OCV and 5 cycles consisting of 1 h at −20 mA cm^–2^ (“on”) followed by 30 min at OCV (“off”) in 0.15 M K_2_SO_4_ (pH 11.5). Leaching measured at (d) the onset of the reaction and (e) at the steady state during the remainder of the 1 h cycle, summarizing the effect of nitrate for both catalysts in a flow-by configuration.

We also evaluated Cu leaching in the absence of nitrate using the flow-by configuration under pulsed “on/off” operation. As in the nitrate-containing flow-by experiments discussed above, a pronounced Cu leaching spike was observed at the reaction onset for both Cu_1_ and Cu_NP_ (Figure 4c). Two key differences emerged when comparing 0 M nitrate and 0.1 M nitrate shown previously (Figure 3b). First, the magnitude of Cu leaching during the initial off-to-on transition was significantly higher in the presence of nitrate for both catalysts. Second, Cu_NP_ leaching remained above 20 μg cm^–2^ for each off-to-on cycle under the no-nitrate, HER-only condition, whereas leaching during successive current transitions progressively decayed under 0.1 M KNO_3_ (Figure 3b). This sustained leaching behavior likely reflects electrolyte-dependent restructuring of the Cu catalyst which will be further discussed below.

To better compare leaching across different conditions, we plotted initial leaching (Cu_onset_, Figure 4d), defined as the leaching measured at the reaction onset, and average leaching ( $\overset{®}{Cu}$ , Figure 4e), defined as the mean leaching during the 1 h reaction cycle, excluding the initial leaching. Cu_onset_ increased by 186% for Cu_1_ and 55% for Cu_NP_ between the no-nitrate and 0.1 M nitrate conditions, consistent with the aforementioned voltage-controlled batch experiments. In marked contrast, $\overset{®}{Cu}$ showed no significant change for Cu_1_ between the two electrolytes, whereas it decreased slightly for Cu_NP_ in the 0.1 M nitrate condition likely due to differences in local pH.

Previous CO_2_RR reports have shown that electron-withdrawing adsorbates, such as *CO, can strengthen adsorbate–metal interactions while weakening metal–metal interactions, facilitating metal dissolution, whereas adsorbed protons exert a weaker effect.^31^ Thus, from an energetic perspective, Cu dissolution reflects a balance between Cu and the support (Cu–N) or Cu–Cu/Cu–O bond strengths, Cu–adsorbate interaction, and the solvation free energy of dissolved Cu species. Given that nitrate alters the leaching behavior, we infer that nitrate or nitrate reduction intermediates similarly compromise the stability of the Cu site by (i) acting as electron-withdrawing species that redistribute charge density and weaken the Cu–N bonds in Cu_1_ or Cu–Cu/Cu–O bonds in Cu_NP_ and/or (ii) stabilizing the leached Cu ion in the electrolyte through solvation or complexation, reducing the energetic barrier for dissolution.^37,41^ The stronger nitrate-concentration dependence observed in both potential- and current-controlled experiments for Cu_1_ indicates that Cu–N bonds in Cu_1_ are more readily perturbed when nitrate species are present at higher concentrations than for Cu–Cu/Cu–O bonding in Cu_NP_.

### *In Situ* XAS Reveals Conformational Changes of Cu_1_ and Cu_NP_

To gain insight into the dynamics of the Cu catalysts during NO_3_RR, we conducted *in situ* XAS experiments to monitor in real time (i) changes in electronic and geometric structures and (ii) Cu leaching under operating conditions in nitrate-containing and nitrate-free electrolytes. Pre-reaction scans at OCV confirmed no X-ray-induced structural damage (Figure S17). We then applied a reductive potential of −0.8 V and collected time-resolved XAS spectra with 1 min temporal resolution.

Under NO_3_RR conditions, Cu_1_ underwent rapid, but reversible, aggregation. Within the first minute after applying the potential, the Cu_1_ white-line intensity sharply decreased, and the resulting spectral shape was maintained throughout 1 h reduction (Figure 5a). Analysis of the EXAFS region indicates the formation of metallic Cu clusters with a CN_Cu–Cu_ of 6.6 (Figure S18 and Table S5). We performed linear combination fitting to track the fractions of single-atom Cu (Cu–N) versus metallic Cu (Cu–Cu) as a function of time (Figure 5b). Returning to OCV post reaction, the as-formed clusters redispersed back to a predominantly single-atom configuration, consistent with prior reports.^15,42,43^ However, post-reaction HAADF-STEM images taken after an 8 h reaction at −0.8 V revealed Cu_1_ redistribution into denser regions (Figure S19), indicating that although the single-atom configuration was largely restored, electrolysis may have altered the spatial distribution of Cu_1_.

**Figure 5:**
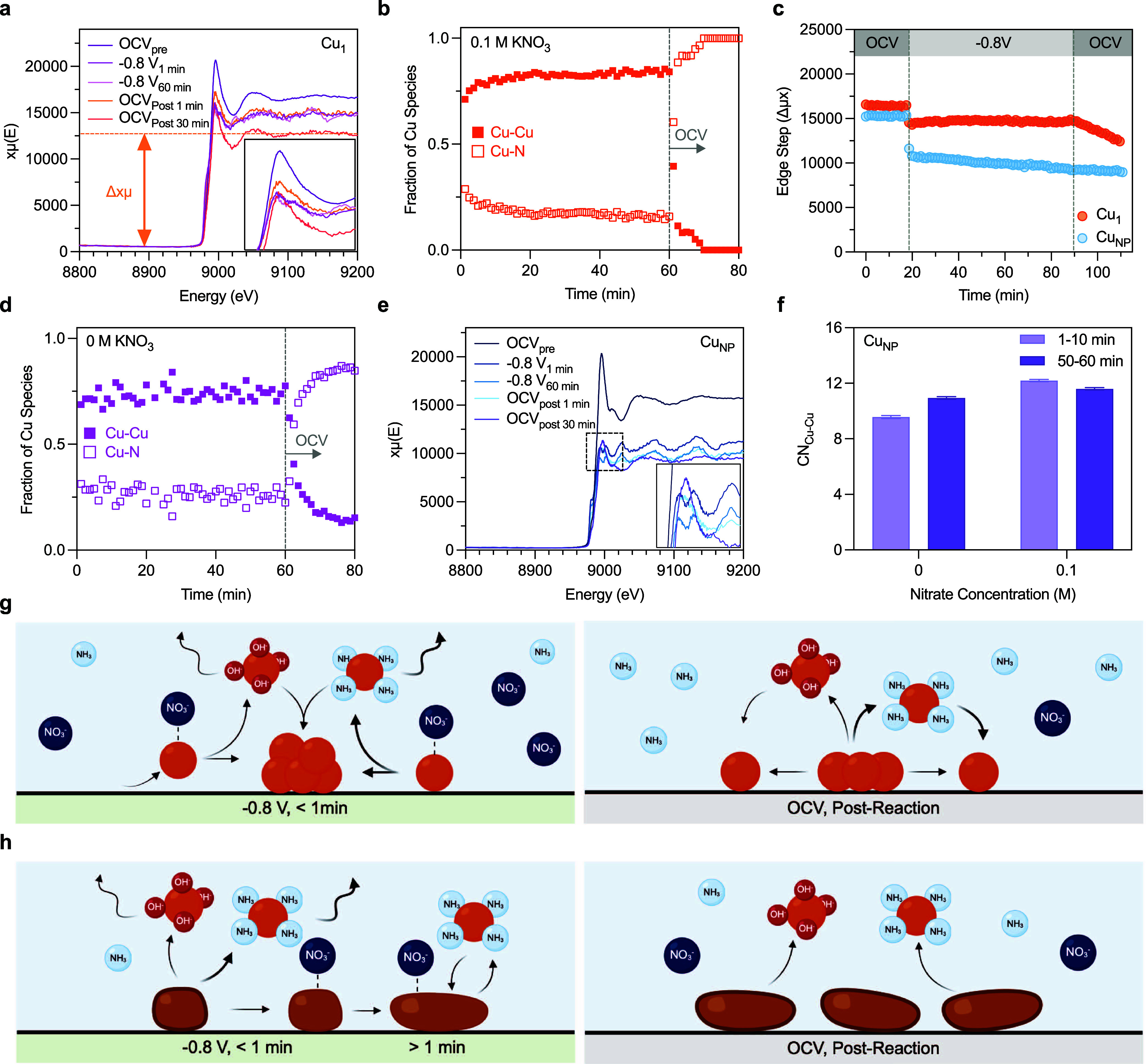
(a) *In situ* XAS spectra of selected time points for reduction of Cu_1_ and (b) linear combination fitting determined time-dependent profile of Cu_1_ at −0.8 V in 0.1 M KNO_3_. (c) Edge step profile during *in situ* XAS for both catalysts. (d) Linear combination fitting determined time-dependent profile of Cu_1_ at −0.8 V in 0 M KNO_3_. (e) Selected time points of *in situ* XAS spectra of Cu_NP_ and (f) coordination number of Cu_NP_ by fitting spectra averaged over the first and last 10 min. Schematic of leaching and reconstruction of (g) Cu_1_ and (h) Cu_NP_ during NO_3_RR at −0.8 V.

We further quantified Cu loss from the electrode based on changes in the XANES edge step, i.e., the difference in the pre- and post-edge normalization lines at E_0_, which scales with the Cu concentration within the sampling area.^44^ At the onset of the applied potential, the edge step decreased, with no further decrease as the potential was held (Figure 5a,c). Upon returning to OCV, the edge step decreased approximately linearly over a subsequent 30 min period, implying continued, gradual Cu loss. These features mirror the spikes in Cu loss at the reaction onset and the slight increase in leaching immediately after returning to the OCV, both observed in flow-by experiments (Figure 3). We cannot unambiguously distinguish whether the observed post-reaction leaching is due to detached clusters or Cu in ionic form. However, the gradual leaching after the OCV, when clustering is largely reversed, suggests that dissolved or weakly bound species formed during the reoxidation of clusters diffuse away from the surface and are likely the source of this measured loss. Together, Cu_1_ structural evolution and edge-step analysis indicate that Cu_1_ restructuring is accompanied by leaching primarily during potential transitions.

*In situ* XAS in the nitrate-free electrolyte further revealed nitrate-dependent differences in restructuring and Cu loss. In the absence of nitrate, Cu_1_ still restructured at potential onset; however, EXAFS fittings at −0.8 V showed higher CN_Cu–Cu_ in the nitrate-containing electrolyte, suggesting that nitrate promotes the mobility of Cu atoms (Figures S18 and S20 and Table S5). Consistently, within the first 5 min, the Cu_1_ fraction decreased and the Cu–Cu fraction increased at a greater magnitude in the nitrate-containing electrolyte (Figure 5b,d). Notably, only ∼80% of the Cu_1_ species were recovered after returning to OCV in the nitrate-free electrolyte. The corresponding edge-step decrease (6%) was substantially smaller than that observed in the presence of nitrate (12%) (Figure S21). These results again support that nitrate enhances Cu_1_ mobility and aggregation during reduction while also enabling more complete redispersion upon reoxidation, potentially by stabilizing dissolved Cu^2+^ species through complexation with NO_3_RR products or intermediates that can act as carriers during reduction/oxidation.^15,45^

Under NO_3_RR conditions, Cu_NP_ underwent structural and Cu content change. The oxide shell of Cu_NP_ was reduced to metallic Cu, with a CN_Cu–Cu_ of 11.6 (Figures 5e and S22 and Table S5). This restructuring coincided with a change in Cu content during NO_3_RR at −0.8 V. At potential onset, the Cu_NP_ edge step dropped by ∼24% (Figure 5c), consistent with the leaching spikes observed at the reaction onset in flow-by experiments (Figure 3). Unlike Cu_1_, Cu_NP_ exhibited continuous Cu loss throughout electrolysis, consistent with dissolution via coexisting soluble Cu­(OH)_2_ species and/or nanoparticle detachment during the reaction.^16^ Upon returning to OCV, the Cu_NP_ structure formed during the applied potential reoxidized (Figure 5e).

Cu_NP_ restructuring and leaching behavior was also affected by the presence of nitrate. Comparison of CN_Cu–Cu_ values obtained by fitting the averaged spectra over the first and last 10 min of the 1 h, −0.8 V reaction reveals opposite trends depending on the electrolyte. In the nitrate-free electrolyte, CN_Cu–Cu_ increased from 9.6 to 10.9 (Figure S23 and Table S5). In the nitrate-containing electrolyte, CN_Cu–Cu_ decreased from 12.2 to 11.6 (Figure 5f). The overall greater CN_Cu–Cu_ in the presence of nitrate indicates that nitrate promotes reconstruction, likely through oxide shell reduction followed by nanoparticle sintering (Figure S22).^14,17^ In contrast, a comparatively lower CN_Cu–Cu_ under the nitrate-free condition may help explain why onset leaching does not decay with increasing cycle number under HER-only conditions; incomplete oxide reduction and/or limited sintering during the initial cycle can enable renewed reconstruction in subsequent cycles, sustaining Cu loss (Figure 4c). Additionally, the Cu_NP_ edge-step drop at potential onset was substantially smaller in HER-only conditions (6.5%; Figure S21). The gradual edge-step drop observed during *in situ* operation is consistent with Cu loss, as exemplified by higher steady-state leaching quantified under the nitrate-free condition (Figure 4e). Overall, these findings indicate that nitrate promotes concurrent Cu restructuring and leaching at the reaction onset for the nanoparticle motif, Cu_NP_.

Across all of the conditions tested in this study, Cu_NP_ exhibited greater leaching than Cu_1_. We postulate that this behavior arises from the reduction of the copper oxide shell to metallic Cu or nanoparticle detachment at the reaction onset.^17,25,26^ In contrast, Cu_1_ did not exhibit spontaneous oxidation upon exposure to an electrolyte. Additionally, single-atom sites are stabilized by strong Cu–N_4_ coordination bonds, which enhance Cu–substrate interactions and increase the energetic barrier of Cu detachment, whereas Cu_NP_ anchorage relies on noncovalent interactions.^46^ Although further investigation is required to fully isolate the pathways and determine underlying mechanisms, these observations underscore the heightened susceptibility of Cu_NP_ to early-stage structural instability. It is noteworthy that although Cu single atoms are deemed unstable due to their tendency to aggregate to minimize surface energy,^13^ the Cu_1_ catalyst studied here displayed greater resistance to leaching than the Cu_NP_ catalyst.

By combining XAS structural and edge-step analyses, we confirm that Cu catalyst restructuring is coupled to leaching. This behavior was observed at the onset of all experiments, independent of the catalyst morphology or electrolyte composition. Therefore, Cu loss can be attributed to copper reduction processes, such as single-atom aggregation or oxide shell reduction. In the presence of nitrate, Cu leaching was exacerbated, likely due to nitrate-derived species lowering the energetic barrier for bond cleavage and surface migration, thereby accelerating structural evolution and metal loss.^48^ For both catalysts, leached Cu may be stabilized at the electrode interface by the electric field and via complexation, redeposited onto the electrode to form larger structures, or transported into the bulk electrolyte (Figure 5g,h).

### Environmental Implications

Understanding how catalyst restructuring translates into material loss is critical to designing durable, safe electrocatalysts. Here, we show that Cu dissolution during NO_3_RR is tightly coupled to dynamic structural evolution and is governed by both catalyst morphology and electrolyte composition: Cu_1_ exhibits comparatively low Cu loss with weak dependence on potential, yet leaching increases with nitrate concentration, whereas Cu_NP_ leaches substantially more and displays stronger sensitivity to both potential and nitrate. Together, these results emphasize that durability cannot be inferred from thermodynamic stability alone and that electrolyte-driven interactions can destabilize Cu active sites, even under nominally reducing conditions.

By correlating leaching measurements with *in situ* XAS, we identify restructuring-driven transitions, especially at the reaction onset, as the dominant window for Cu loss. Structural changes associated with reduction (including single-atom aggregation for Cu_1_ and oxide shell reduction/sintering for Cu_NP_) coincide with the largest leaching events, and nitrate further amplifies these processes while promoting redispersion of Cu_1_ upon returning to OCV. We further demonstrate that the XANES edge step provides a probe of the Cu content, enabling a quantitative linkage between catalyst evolution and material loss under *in situ* conditions.

Collectively, these findings highlight a central challenge for nano- and sub-nanometer electrocatalysts: while such materials offer high intrinsic activity, their propensity for restructuring under reaction conditions can result in material loss, which raises long-term durability concerns. Our results underscore the need to consider how operating conditions, such as electrolyte composition and intermittent operation, impact catalyst working structure and material loss, in addition to reaction mechanisms and selectivity. More broadly, they demonstrate the importance of *in situ* characterization for capturing dynamic structural changes that may govern catalyst lifetime and metal contamination of treated water.

Future work should focus on evaluating catalyst dissolution under realistic electrolytes and developing restructuring mitigation strategies to minimize copper loss and ensure long-term durability under relevant electrochemical NO_3_RR conditions. For single-atom catalysts, such strategies may include modifying the coordinating atom identity to strengthen metal–substrate bonding.^49,50^ In nanoparticles, increasing substrate defect density has been shown to suppress CuO_x_ NP restructuring.^20^ Additionally, higher ionomer concentrations in catalyst inks have been shown to mitigate nanoparticle detachment during CO_2_RR and reduce noble metal dissolution during anodic reactions.^20,51^ While elevated concentrations of hydroxide ions may promote Cu dissolution during operation, electrolyte engineering to modulate surface pH warrants careful investigation as certain buffering anions, such as bicarbonate, can poison catalysts or alter reaction pathways.^52,53^ These strategies should be evaluated using *in situ* characterization techniques and flow-through systems to better understand their effects, inform design considerations, and ultimately enable effective and safe implementation.

## Supplementary Material



## Data Availability

All relevant data that support the findings of this study are available from the corresponding author upon reasonable request.

## Notes

Methods, HAADF-STEM images, nitrate reduction performance, electrochemical characterization, and XAS spectra and fits (PDF)

## References

[1] Liu X., Liu J., Chen A., Yang S., Jiang L., Sui Y. (2025). 99.7% Faradaic
Efficiency in Nitrate Reduction Enabled by Defect-Rich Copper Nanoparticles. ACS Appl. Nano Mater..

[2] Huang Y., He C., Cheng C., Han S., He M., Wang Y., Meng N., Zhang B., Lu Q., Yu Y. (2023). Pulsed Electroreduction
of Low-Concentration Nitrate to Ammonia. Nat.
Commun..

[3] Fan J., Wu Y., Arrazolo L. K., Yin K., Jin S., Gao Y., Wu X., Wu Z., Kim J.-H. (2026). Pulse-Engineered Ion Redistribution
Suppresses Cation Interference in Electrocatalytic Nitrate Reduction. Environ. Sci. Technol..

[4] Du C., Lu S., Wang J., Wang X., Wang M., Fruehwald H. M., Wang L., Zhang B., Guo T., Mills J. P., Wei W., Chen Z., Teng Y., Zhang J., Sun C.-J., Zhou H., Smith R. D. L., Kendall B., Henkelman G., Wu Y. A. (2023). Selectively Reducing Nitrate into NH_3_ in Neutral Media
by PdCu Single-Atom Alloy Electrocatalysis. ACS Catal..

[5] Wang Y., Zhou W., Jia R., Yu Y., Zhang B. (2020). Unveiling
the Activity Origin of a Copper-Based Electrocatalyst for Selective
Nitrate Reduction to Ammonia. Angew. Chem.,
Int. Ed..

[6] Jung W., Hwang Y. J. (2021). Material
Strategies in the Electrochemical Nitrate
Reduction Reaction to Ammonia Production. Mater.
Chem. Front..

[7] Zhang K., Liu Y., Pan Z., Xia Q., Huo X., Esan O. C., Zhang X., An L. (2024). Cu-Based Catalysts
for Electrocatalytic
Nitrate Reduction to Ammonia: Fundamentals and Recent Advances. EES Catal..

[8] Liu X., Xiang T., Liu J., Huang B., Yang Y., Qu X., Xiong W., Cheng M., Jiang G., Liu Y., Zhou C. (2026). Ag Single Atoms Boosting Water Dissociation on Cu Nanowires for Efficient
H*-Mediated Nitrate Reduction at Ultra-Low Concentrations With Ammonia
Recovery. Adv. Funct. Mater..

[9] Liu X., Xiang T., Liang Y., Zhou X., Wang Z., Liu J., Cheng M., Zhou C., Yang Y. (2024). When Electrocatalytic
Nitrate Reduction Meets Copper-Based Atomic Site Catalysts. J. Mater. Chem. A.

[10] Amirbeigiarab R., Tian J., Herzog A., Qiu C., Bergmann A., Roldan Cuenya B., Magnussen O. M. (2023). Atomic-Scale Surface Restructuring
of Copper Electrodes under CO_2_ Electroreduction Conditions. Nat. Catal..

[11] Tomc B., Bele M., Plut M., Kostelec M., Popović S., Nazrulla M. A., Ruiz-Zepeda F., Kamšek A. R., Šala M., Elbataioui A., Rafailović L. D., Pissolitto Y. B., Trivinho-Strixino F., Stępniowski W. J., Suhadolnik L., Hodnik N. (2025). Recognizing the Universality of Copper
Reconstruction Via Dissolution-Redeposition at the Onset of CO_2_ Reduction. J. Phys. Chem. Lett..

[12] Yang X.-F., Wang A., Qiao B., Li J., Liu J., Zhang T. (2013). Single-Atom Catalysts: A New Frontier
in Heterogeneous Catalysis. Acc. Chem. Res..

[13] Rigby K., Kim J.-H. (2023). Deciphering the
Issue of Single-Atom Catalyst Stability. Curr.
Opin. Chem. Eng..

[14] Mandal L., Yang K. R., Motapothula M. R., Ren D., Lobaccaro P., Patra A., Sherburne M., Batista V. S., Yeo B. S., Ager J. W., Martin J., Venkatesan T. (2018). Investigating
the Role of Copper Oxide in Electrochemical CO_2_ Reduction
in Real Time. ACS Appl. Mater. Interfaces.

[15] Yang J., Qi H., Li A., Liu X., Yang X., Zhang S., Zhao Q., Jiang Q., Su Y., Zhang L., Li J.-F., Tian Z.-Q., Liu W., Wang A., Zhang T. (2022). Potential-Driven Restructuring of
Cu Single Atoms to Nanoparticles
for Boosting the Electrochemical Reduction of Nitrate to Ammonia. J. Am. Chem. Soc..

[16] Yoon A., Bai L., Yang F., Franco F., Zhan C., Rüscher M., Timoshenko J., Pratsch C., Werner S., Jeon H. S., Monteiro M. C. de O., Chee S. W., Roldan Cuenya B. (2025). Revealing
Catalyst Restructuring and Composition during Nitrate Electroreduction
through Correlated Operando Microscopy and Spectroscopy. Nat. Mater..

[17] Vavra J., Shen T.-H., Stoian D., Tileli V., Buonsanti R. (2021). Real-Time
Monitoring Reveals Dissolution/Redeposition Mechanism in Copper Nanocatalysts
during the Initial Stages of the CO_2_ Reduction Reaction. Angew. Chem., Int. Ed..

[18] Osowiecki W. T., Nussbaum J. J., Kamat G. A., Katsoukis G., Ledendecker M., Frei H., Bell A. T., Alivisatos A. P. (2019). Factors
and Dynamics of Cu Nanocrystal Reconstruction under CO_2_ Reduction. ACS Appl. Energy Mater..

[19] Bai L., Franco F., Timoshenko J., Rettenmaier C., Scholten F., Jeon H. S., Yoon A., Rüscher M., Herzog A., Haase F. T., Kühl S., Chee S. W., Bergmann A., Beatriz R. C. (2024). Electrocatalytic
Nitrate and Nitrite Reduction toward Ammonia Using Cu_2_O
Nanocubes: Active Species and Reaction Mechanisms. J. Am. Chem. Soc..

[20] Lee S. H., Avilés Acosta J. E., Lee D., Larson D. M., Li H., Chen J., Lee J., Erdem E., Lee D. U., Blair S. J., Gallo A., Zheng H., Nielander A. C., Tassone C. J., Jaramillo T. F., Drisdell W. S. (2025). Structural Transformation
and Degradation of Cu Oxide Nanocatalysts during Electrochemical CO_2_ Reduction. J. Am. Chem. Soc..

[21] Cherevko S., Zeradjanin A. R., Topalov A. A., Kulyk N., Katsounaros I., Mayrhofer K. J. J. (2014). Dissolution of Noble Metals during Oxygen Evolution
in Acidic Media. ChemCatChem.

[22] Geiger S., Kasian O., Ledendecker M., Pizzutilo E., Mingers A. M., Fu W. T., Diaz-Morales O., Li Z., Oellers T., Fruchter L., Ludwig A., Mayrhofer K. J. J., Koper M. T. M., Cherevko S. (2018). The Stability Number as a Metric
for Electrocatalyst Stability Benchmarking. Nat. Catal..

[23] Reier T., Oezaslan M., Strasser P. (2012). Electrocatalytic
Oxygen Evolution
Reaction (OER) on Ru, Ir, and Pt Catalysts: A Comparative Study of
Nanoparticles and Bulk Materials. ACS Catal..

[24] Cao L., Luo Q., Chen J., Wang L., Lin Y., Wang H., Liu X., Shen X., Zhang W., Liu W., Qi Z., Jiang Z., Yang J., Yao T. (2019). Dynamic Oxygen Adsorption
on Single-Atomic Ruthenium Catalyst with High Performance for Acidic
Oxygen Evolution Reaction. Nat. Commun..

[25] Yan K., Lee S.-W., Yap K. M. K., Mule A. S., Hannagan R. T., Matthews J. E., Kamat G. A., Lee D. U., Stevens M. B., Nielander A. C., Jaramillo T. F. (2025). On-Line Inductively Coupled Plasma
Mass Spectrometry Reveals Material Degradation Dynamics of Au and
Cu Catalysts during Electrochemical CO_2_ Reduction. J. Am. Chem. Soc..

[26] Speck F. D., Cherevko S. (2020). Electrochemical Copper Dissolution: A Benchmark for
Stable CO_2_ Reduction on Copper Electrocatalysts. Electrochem. Commun..

[27] Martini A., Hursán D., Timoshenko J., Rüscher M., Haase F., Rettenmaier C., Ortega E., Etxebarria A., Roldan Cuenya B. (2023). Tracking the
Evolution of Single-Atom Catalysts for
the CO_2_ Electrocatalytic Reduction Using *Operando* X-Ray Absorption Spectroscopy and Machine Learning. J. Am. Chem. Soc..

[28] Martini A., Timoshenko J., Rüscher M., Hursán D., Monteiro M. C. O., Liberra E., Roldan Cuenya B. (2024). Revealing
the Structure of the Active Sites for the Electrocatalytic CO_2_ Reduction to CO over Co Single Atom Catalysts Using *Operando* XANES and Machine Learning. J. Synchrotron Radiat..

[29] Zhang P., Chen H.-C., Zhu H., Chen K., Li T., Zhao Y., Li J., Hu R., Huang S., Zhu W., Liu Y., Pan Y. (2024). Inter-Site Structural Heterogeneity
Induction of Single Atom Fe Catalysts for Robust Oxygen Reduction. Nat. Commun..

[30] Yang H., Shang L., Zhang Q., Shi R., Waterhouse G. I. N., Gu L., Zhang T. (2019). A Universal
Ligand Mediated Method
for Large Scale Synthesis of Transition Metal Single Atom Catalysts. Nat. Commun..

[31] Wu X., Nazemi M., Gupta S., Chismar A., Hong K., Jacobs H., Zhang W., Rigby K., Hedtke T., Wang Q., Stavitski E., Wong M. S., Muhich C., Kim J.-H. (2023). Contrasting Capability
of Single Atom Palladium for
Thermocatalytic versus Electrocatalytic Nitrate Reduction Reaction. ACS Catal..

[32] Leshchev D., Rakitin M., Luvizotto B., Kadyrov R., Ravel B., Attenkofer K., Stavitski E. (2022). The Inner Shell Spectroscopy Beamline
at NSLS-II: A Facility for *in Situ* and *Operando* X-Ray Absorption Spectroscopy for Materials Research. J. Synchrotron Radiat..

[33] Li P., Jin Z., Fang Z., Yu G. (2021). A Single-Site Iron
Catalyst with
Preoccupied Active Centers That Achieves Selective Ammonia Electrosynthesis
from Nitrate. Energy Environ. Sci..

[34] Zhu T., Chen Q., Liao P., Duan W., Liang S., Yan Z., Feng C. (2020). Single-Atom
Cu Catalysts for Enhanced Electrocatalytic
Nitrate Reduction with Significant Alleviation of Nitrite Production. Small.

[35] Vavra J., Ramona G. P. L., Dattila F., Kormányos A., Priamushko T., Albertini P. P., Loiudice A., Cherevko S., Lopéz N., Buonsanti R. (2024). Solution-Based Cu^+^ Transient
Species Mediate the Reconstruction of Copper Electrocatalysts for
CO_2_ Reduction. Nat. Catal..

[36] Casebolt R., Levine K., Suntivich J., Hanrath T. (2021). Pulse Check: Potential
Opportunities in Pulsed Electrochemical CO_2_ Reduction. Joule.

[37] Kim C., Weng L.-C., Bell A. T. (2020). Impact of Pulsed Electrochemical
Reduction of CO_2_ on the Formation of C_2+_ Products
over Cu. ACS Catal..

[38] Samu A. A., Kormányos A., Kecsenovity E., Szilágyi N., Endrődi B., Janáky C. (2022). Intermittent
Operation of CO_2_ Electrolyzers at Industrially Relevant
Current Densities. ACS Energy Lett..

[39] Nguyen T. N., Chen Z., Zeraati A. S., Shiran H. S., Sadaf S. Md., Kibria M. G., Sargent E. H., Dinh C.-T. (2022). Catalyst Regeneration
via Chemical Oxidation Enables Long-Term Electrochemical Carbon Dioxide
Reduction. J. Am. Chem. Soc..

[40] Feng Y., Huang X., Wu Z., Wang H., Zuo K., Li Q. (2024). Polarity Modulation Enhances Electrocatalytic Reduction
of Nitrate
by Iron Nanocatalysts. ACS EST Eng..

[41] Xiang T., Liu X., Wang Z., Zeng Y., Deng J., Xiong W., Cheng M., Liu J., Zhou C., Yang Y. (2025). Boosting Active
Hydrogen Generation via Ruthenium Single Atoms for Efficient Electrocatalytic
Nitrate Reduction to Ammonia. Appl. Catal. B
Environ. Energy.

[42] Wei X., Liu Y., Zhu X., Bo S., Xiao L., Chen C., Nga T. T. T., He Y., Qiu M., Xie C., Wang D., Liu Q., Dong F., Dong C.-L., Fu X.-Z., Wang S. (2023). Dynamic Reconstitution
Between Copper
Single Atoms and Clusters for Electrocatalytic Urea Synthesis. Adv. Mater..

[43] Bai X., Zhao X., Zhang Y., Ling C., Zhou Y., Wang J., Liu Y. (2022). Dynamic Stability
of Copper Single-Atom
Catalysts under Working Conditions. J. Am. Chem.
Soc..

[44] Leri A. C., Ravel B. (2014). Sample Thickness and Quantitative Concentration Measurements in Br
K-Edge XANES Spectroscopy of Organic Materials. J. Synchrotron Radiat..

[45] Schröder D., Schwarz H., Wu J., Wesdemiotis C. (2001). Long-Lived
Dications of Cu­(H_2_O)^2+^ and Cu­(NH_3_)^2+^ Do Exist!. Chem. Phys. Lett..

[46] Xin L., Yang F., Rasouli S., Qiu Y., Li Z.-F., Uzunoglu A., Sun C.-J., Liu Y., Ferreira P., Li W., Ren Y., Stanciu L. A., Xie J. (2016). Understanding Pt Nanoparticle
Anchoring on Graphene Supports through Surface Functionalization. ACS Catal..

[48] Li F., Medvedeva X. V., Medvedev J. J., Khairullina E., Engelhardt H., Chandrasekar S., Guo Y., Jin J., Lee A., Thérien-Aubin H., Ahmed A., Pang Y., Klinkova A. (2021). Interplay of Electrochemical and Electrical Effects
Induces Structural Transformations in Electrocatalysts. Nat. Catal..

[49] Yao Y., Huang Z., Xie P., Wu L., Ma L., Li T., Pang Z., Jiao M., Liang Z., Gao J., He Y., Kline D. J., Zachariah M. R., Wang C., Lu J., Wu T., Li T., Wang C., Shahbazian-Yassar R., Hu L. (2019). High Temperature Shockwave Stabilized Single Atoms. Nat. Nanotechnol..

[50] Wang L., Zhu C., Xu M., Zhao C., Gu J., Cao L., Zhang X., Sun Z., Wei S., Zhou W., Li W.-X., Lu J. (2021). Boosting Activity and
Stability of
Metal Single-Atom Catalysts via Regulation of Coordination Number
and Local Composition. J. Am. Chem. Soc..

[51] Zlatar M., Simon C., Przybysz J. M., Rodríguez M. G., Katsman J., Ayoub M., Khalakhan I., Cherevko S. (2025). Effects of Loading and Nafion Content on the Activity
and Stability of Iridium Oxygen Evolution Reaction Catalysts. J. Electrochem. Soc..

[52] Pincus L. N., Rudel H. E., Petrović P. V., Gupta S., Westerhoff P., Muhich C. L., Zimmerman J. B. (2020). Exploring the Mechanisms of Selectivity
for Environmentally Significant Oxo-Anion Removal during Water Treatment:
A Review of Common Competing Oxo-Anions and Tools for Quantifying
Selective Adsorption. Environ. Sci. Technol..

[53] Shahaf Y., Slot T. K., Avidan S., Dick J. E., Eisenberg D. (2025). Buffer Effects
on Nitrite Reduction Electrocatalysis. ACS Catal..

