# Supplementary material for: Reconstruction and Dissolution of Copper Catalysts during Electrocatalytic Nitrate Reduction
Source: Environ Sci Technol. 2026 Jun 16;60(25):18355–65. doi: 10.1021/acs.est.6c04463 (PMC13326715; doi:10.1021/acs.est.6c04463)
Supplement: Supplementary file 1 [file es6c04463_si_001.pdf]

Supplemental Information

Reconstruction and Dissolution of Copper Catalysts During Electrocatalytic Nitrate Reduction

Leslie K. Arrazolo[‡,1], Kali Rigby[‡,1], Jorge Moncada[2], Eli Stavitski[2], Jae-Hong Kim[1*]

[1]Department of Chemical and Environmental Engineering, Yale University, New Haven, Connecticut 06510, USA
[2]National Synchrotron Light Source-II, Brookhaven National Laboratory, Upton, New York 11973, USA

31 Pages, 3 Texts, 23 Figures, 5 Tables

*Corresponding author: jaehong.kim@yale.edu

Contents

Text S1. Cu leaching calculations.

Text S2. Flow-by Cu leaching calculations.

Text S3. S-number calculations

Figure S1. Cu_1 electric double layer capacitance before and after NO_3RR reactions.

Figure S2. Cu_1 cyclic voltammetry curves.

Figure S3. Cu_{NP} electric double layer capacitance before and after NO_3RR reactions.

Figure S4. Cu_{NP} cyclic voltammetry curves.

Figure S5. Electric double layer capacitance of graphene.

Figure S6. HAADF-STEM images and EELS spectra.

Figure S7. *Ex situ* Cu_1 EXAFS.

Figure S8. *Ex situ* Cu_{NP} EXAFS.

Figure S9. LSV of Cu_1 and Cu_{NP}.

Figure S10. Batch NO_3RR performance.

Figure S11. Batch Cu leaching profiles at -0.4 V and -0.6 V.

Figure S12. Stability numbers at -0.4 V and -0.6 V.

Figure S13. Flow-by NO_3RR performance.

Figure S14. NO_3RR and nitrate-free flow-by Cu leaching profiles.

Figure S15. Pulsing flow-by NO_3RR performance.

Figure S16. Copper leaching as a function of nitrate concentration in batch operation.

Figure S17. *In situ* XANES spectra at OCV.

Figure S18. *In situ* Cu_1 EXAFS during NO_3RR.

Figure S19. Post-reaction HAADF-STEM catalyst images.

Figure S20. *In situ* Cu_1 EXAFS during HER.

Figure S21. Time evolution of edge step during in situ XAS during HER.

Figure S22. *In situ* Cu_{NP} EXAFS during NO_3RR.

Figure S23. *In situ* Cu_{NP} EXAFS during HER.

Table S1. *Ex situ* FT-EXAFS best-fit parameters.

Table S2. Batch Cu leaching as a percentage of initial Cu loading.

Table S3. Flow-by Cu leaching as a percentage of initial Cu loading.

Table S4. Conductivity of electrolyte solutions.

Table S5. *In situ* FT-EXAFS best-fit parameters.

Text S1. Cu leaching calculations

$$\text{Cu Leached } [\mu\text{g cm}^{-2}] = \frac{ppb_{Cu} \times V_{Batch}}{ECSA}$$

Text S2. Flow-by Cu leaching calculations

Volume of sample collected from cell outlet, V_{sample} = 0.8 mL

Cu ppb (ppb_{Cu}) is the concentration of Cu in the sample collected.

Time for which sample was collected, t = 8 min

$$\text{Cu Leached } [\mu\text{g cm}^{-2}\text{ min}^{-1}] = \frac{ppb_{Cu} \times V_{sample}}{ECSA \times t}$$

Text S3. S-number calculations

Molar mass of Cu (m_{Cu}) = 63.55 g mol^{-1}

$$\text{S-Number} = mol_{NH_3} L^{-1} \times \left[\frac{ppb_{Cu}}{10^6 \times m_{Cu}}\right]^{-1}$$

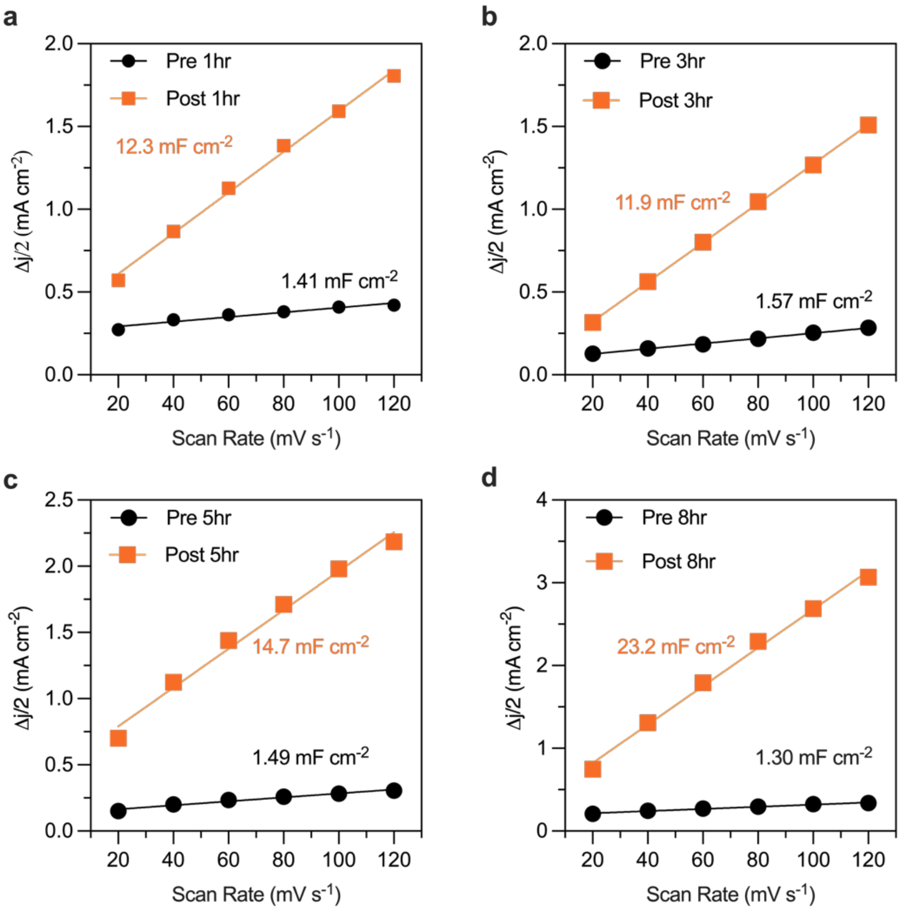

Figure S1. The electric double layer capacitance (C_{dl}) of Cu_1 before and after NO_3RR of various durations in 0.1 M KNO_3 + 0.1 M K_2SO_4, pH 11.5 on a glassy carbon electrode. Data points are shown as markers, and the solid lines represent linear regressions. The C_{dl} increases markedly after 1, 3, 5, and 8 h of reaction at −0.8 V, indicating the generation of additional active sites under prolonged reductive conditions. The electrochemical surface area was calculated by dividing the measured C_{dl} by the C_{dl} of bare graphene and multiplying by the geometric area.

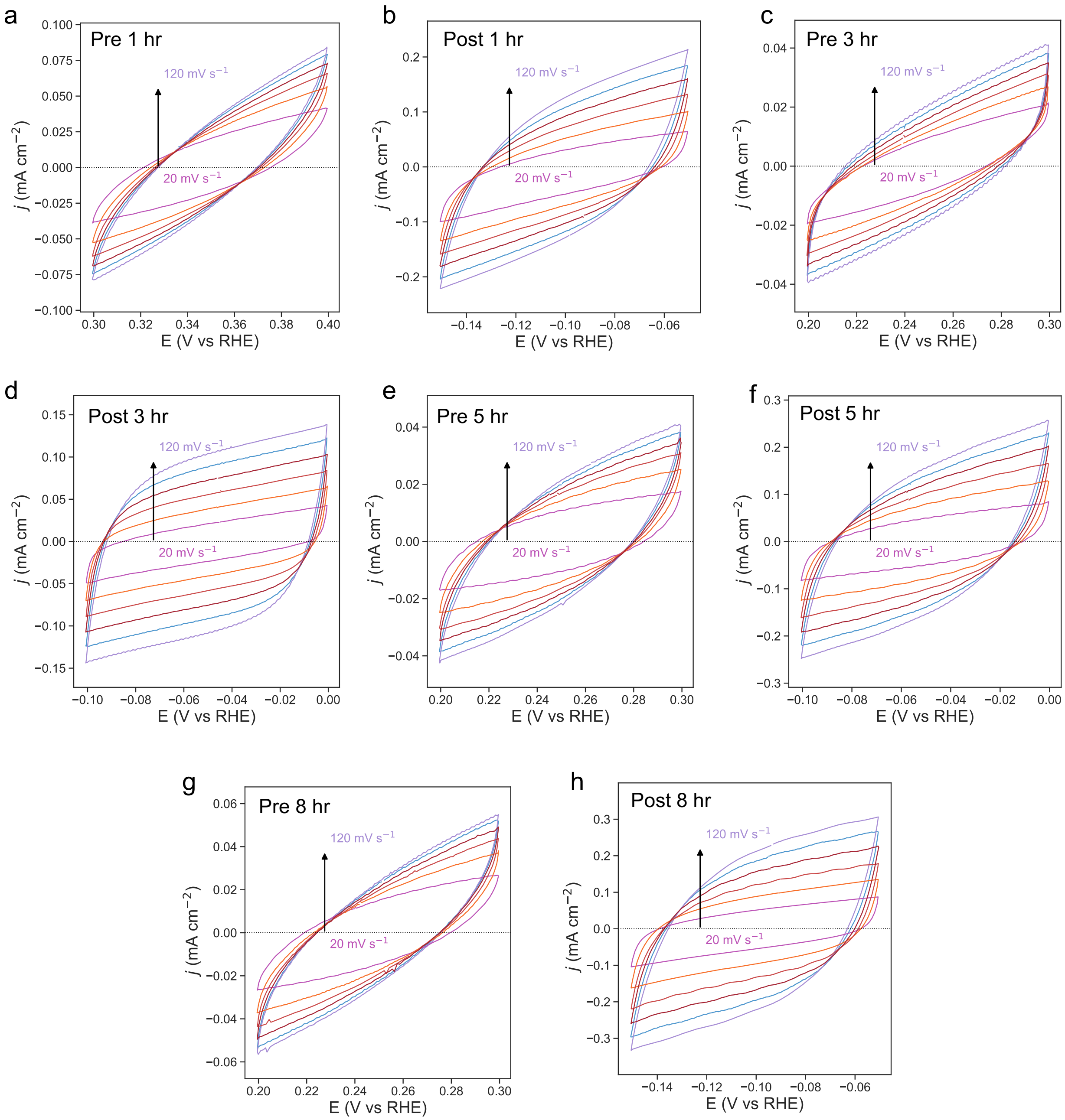

Figure S2. Cyclic voltammetry curves corresponding the C_{dl} measurements of Cu_1 before and after reactions of varying lengths at –0.8 V vs RHE in 0.1 M KNO_3 + 0.1 M K_2SO_4, pH 11.5 on a glassy carbon electrode.

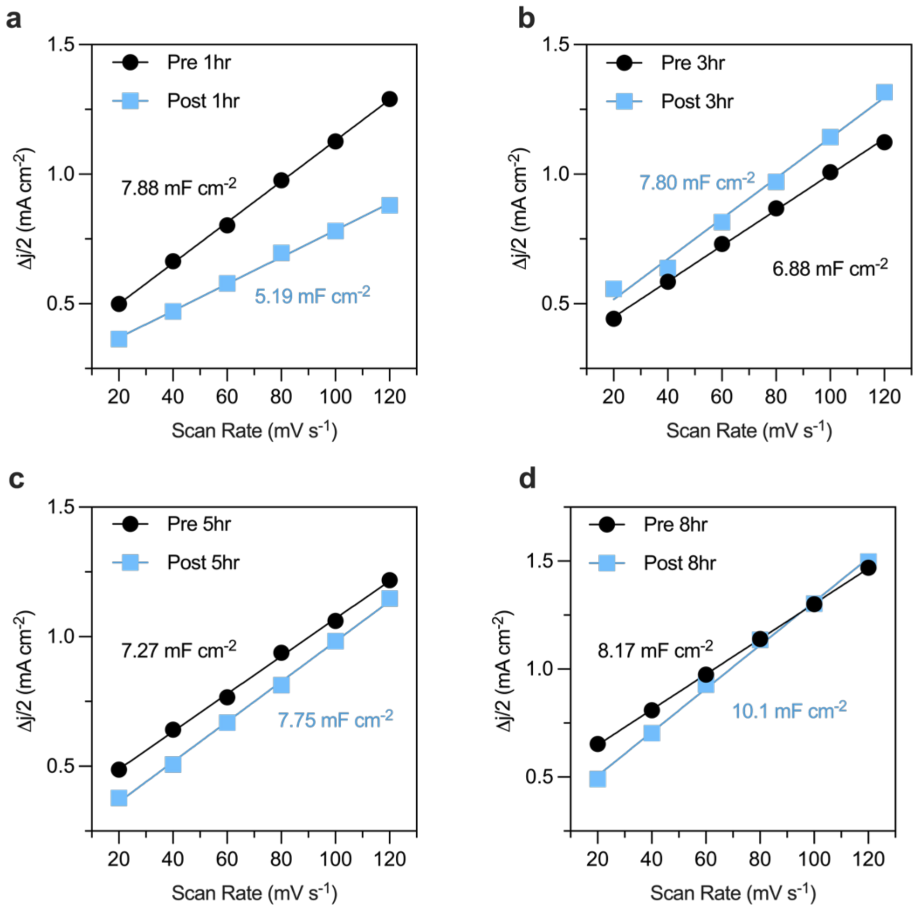

Figure S3. C_{dl} of Cu_{NP} before and after NO_3RR of various durations in 0.1 M KNO_3 + 0.1 M K_2SO_4, pH 11.5 on a glassy carbon electrode. Data points are shown as markers, and the solid lines represent linear regressions. The C_{dl} increases markedly after 3, 5, and 8 h of reaction at −0.8 V, indicating the generation of additional active sites under prolonged reductive conditions. The C_{dl} decreases after 1 h of reaction, but for reactions 3 h or greater, the C_{dl} increases, indicating greater active area after longer reactions.

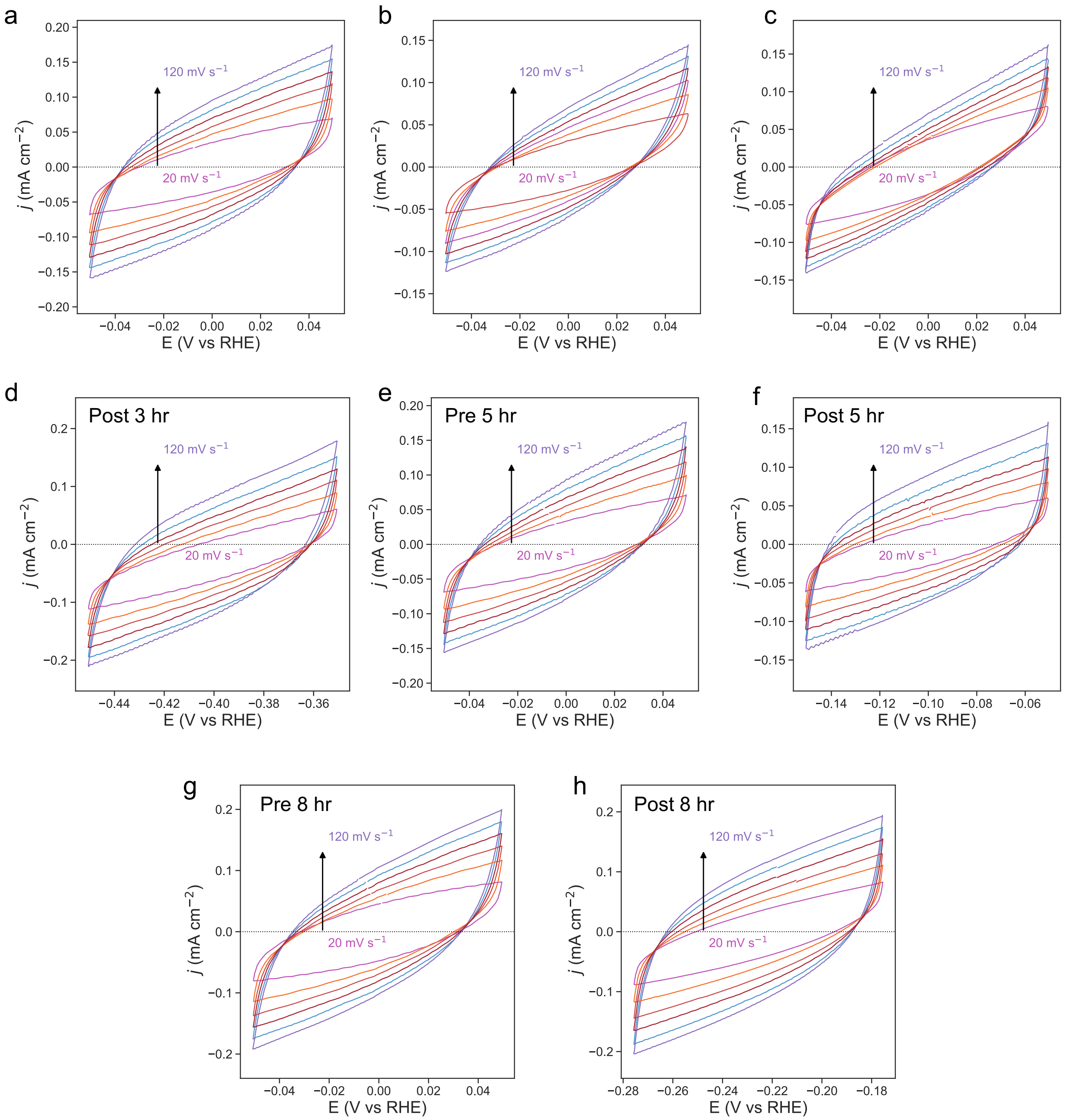

Figure S4. Cyclic voltammetry curves corresponding the C_{dl} measurements of Cu_{NP} before and after reactions of varying lengths at –0.8 V vs RHE in 0.1 M KNO_3 + 0.1 M K_2SO_4, pH 11.5 on a glassy carbon electrode.

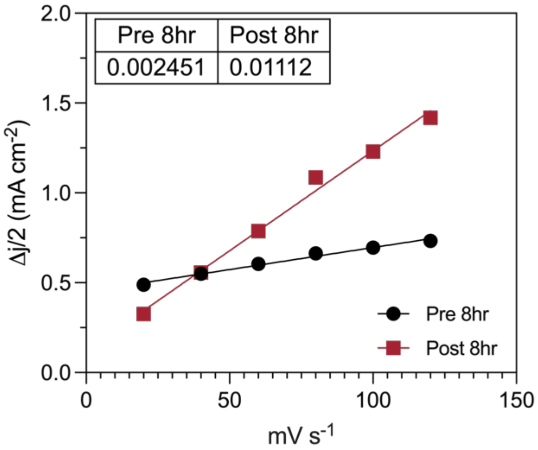

Figure S5. C_{dl} of bare graphene substrate before and after 8 h reaction in 0.1 M KNO_3 + 0.1 M K_2SO_4, pH 11.5 on a glassy carbon electrode. After the reaction, the C_{dl} increases indicating enhanced electrochemical activity of the substrate under reductive conditions.

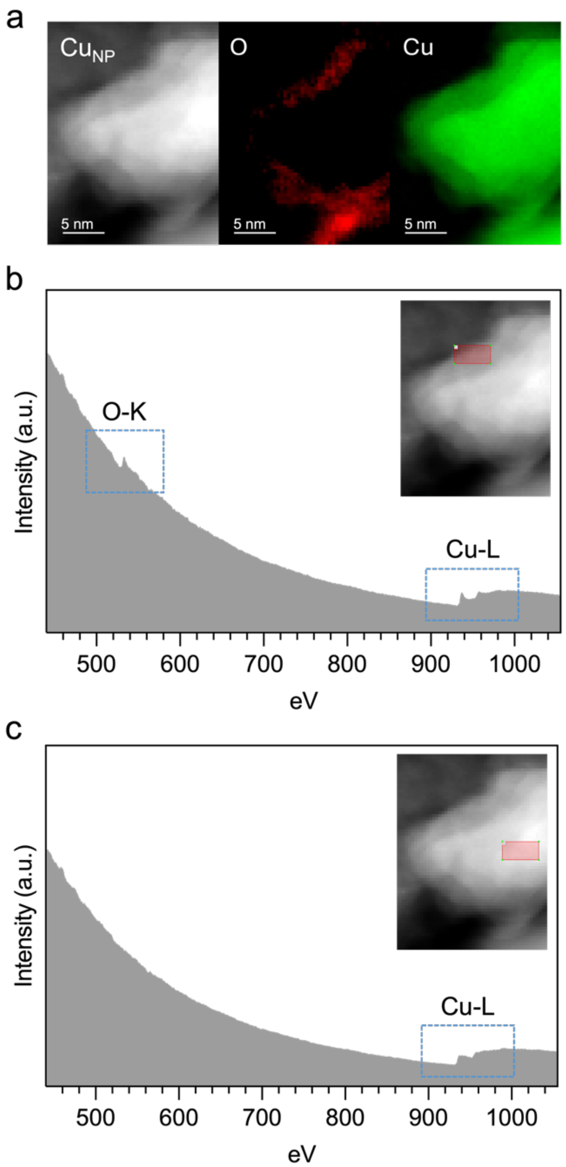

Figure S6. (a) HAADF-STEM image of pristine Cu$_{NP}$ and corresponding EELS elemental maps of O and Cu. EELS spectra of the O K- and Cu L-edges extracted from the regions highlighted in the HAADF-STEM image (red boxes), corresponding to the nanoparticle (b) copper oxide shell and (c) metallic Cu core.

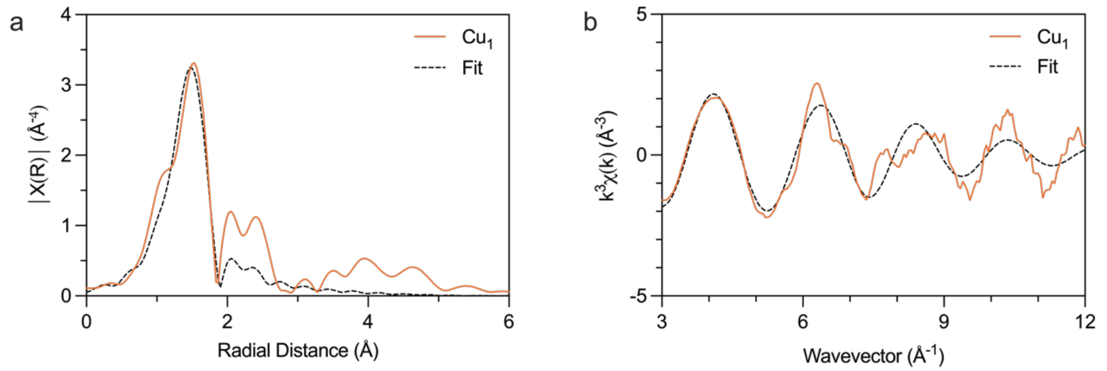

Figure S7. EXAFS fits of Cu_1 measured *ex situ*: (a) R-space and (b) k^3-space. R-space fit ranged from 1-2 Å and k-space fit ranged from 3-12 $Å^{-1}$.

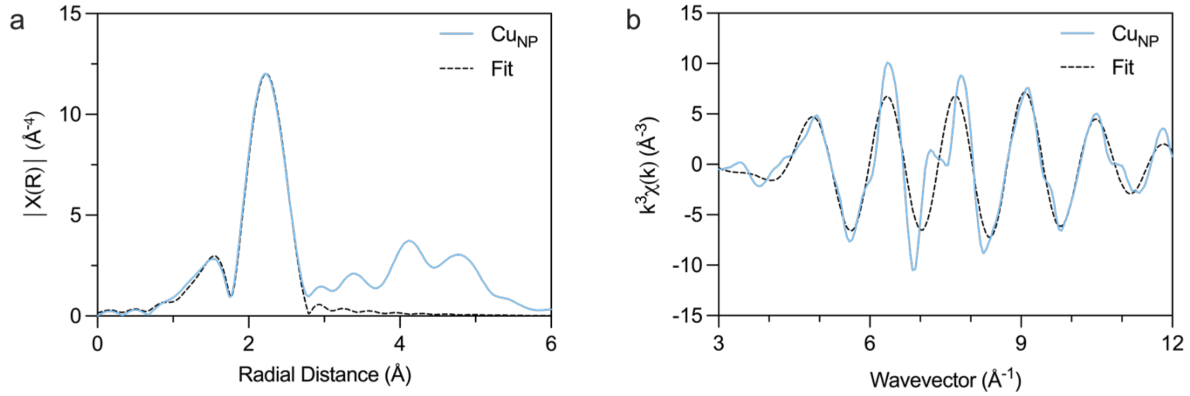

Figure S8. EXAFS fits of Cu$_{NP}$ measured *ex situ*: (a) R-space and (b) k^3-space. R-space fit ranged from 1-3 Å and k-space fit ranged from 3-12 Å^{-1}.

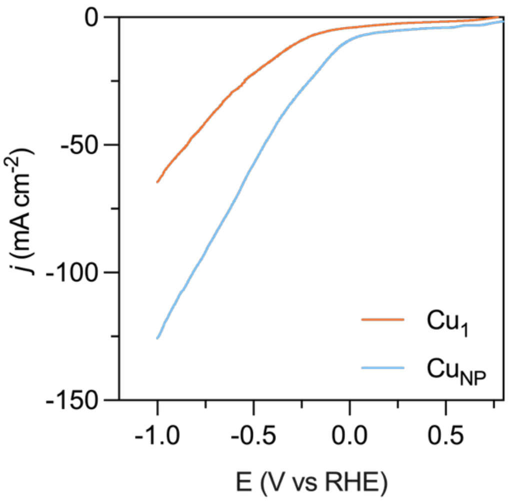

Figure S9. Linear sweep voltammetry (LSV) of Cu_1 and Cu_{NP} in 0.1 M KNO_3 + 0.1 M K_2SO_4 at pH 11.5.

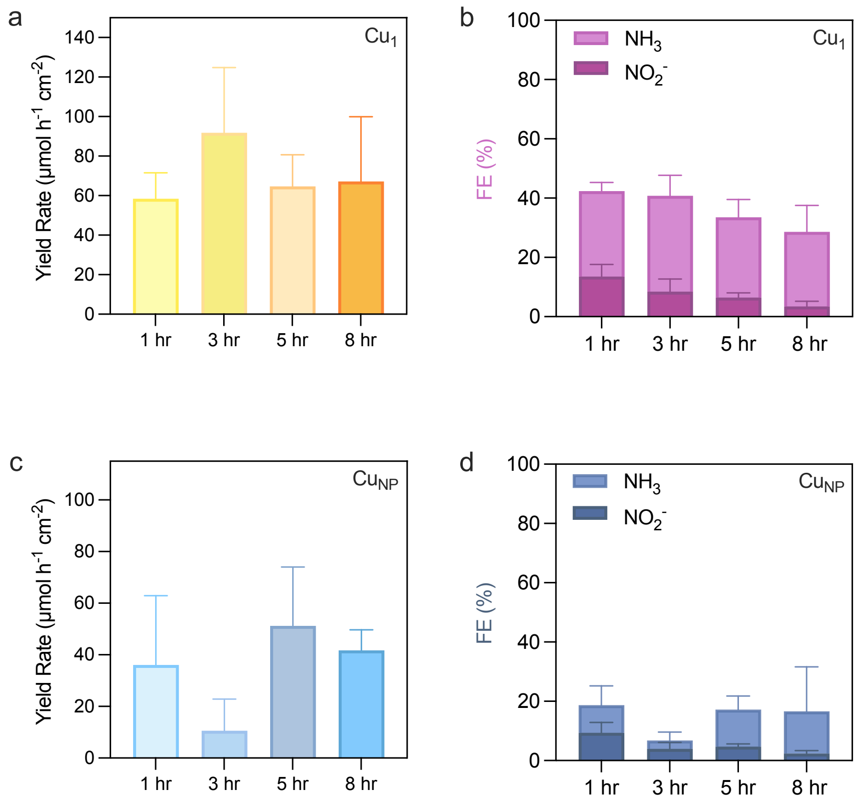

Figure S10. Nitrate conversion and Faradaic efficiency for (a,b) Cu_1 and (c,d) Cu_{NP} at –0.8 V for 1, 3, 5, and 8 h of electrolysis. Conditions: 0.1 M KNO_3 + 0.1 M K_2SO_4, pH 11.5.

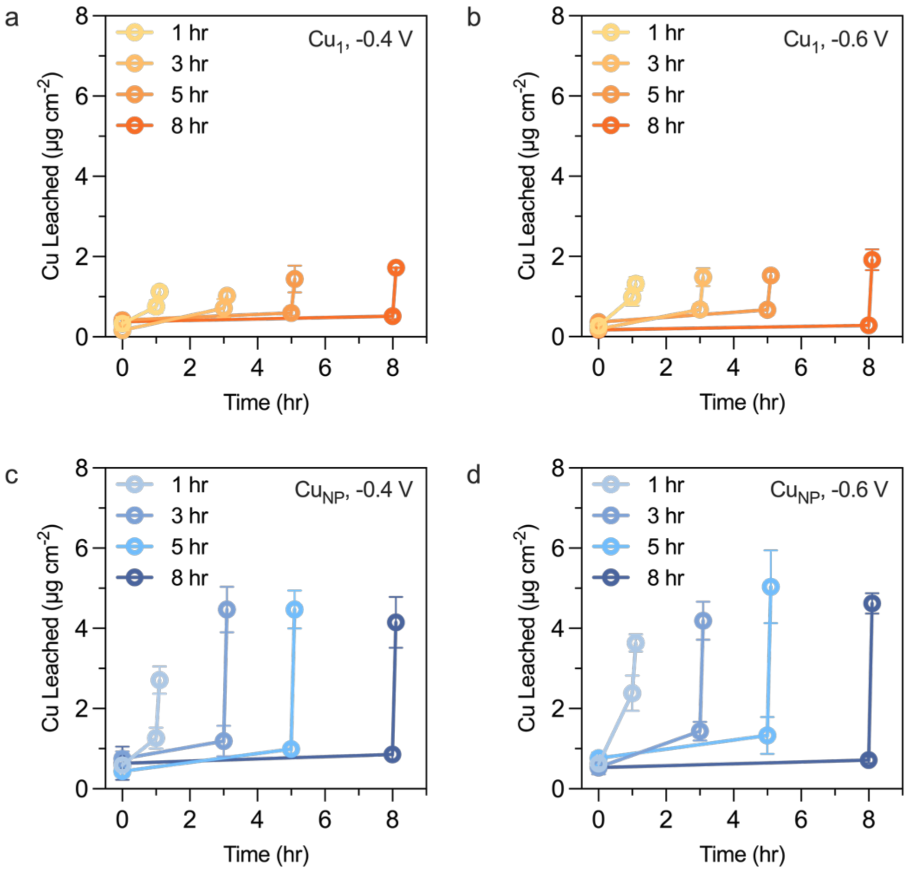

Figure S11. Copper leaching profiles for (a,b) Cu_1 and (c,d) Cu_{NP} at –0.4 V and –0.6 V during nitrate reduction for 1, 3, 5, and 8 h. Samples were collected after 30 minutes at OCV, at the end of electrolysis, and 5 min post-reaction at OCV. Conditions: 0.1 M KNO_3 + 0.1 M K_2SO_4, pH 11.5.

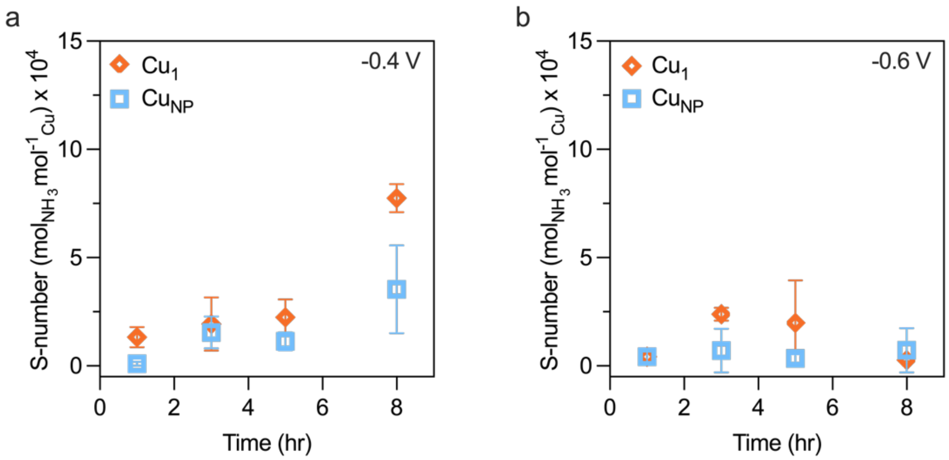

Figure S12. Stability numbers (S-number) for Cu_1 and Cu_{NP} as a function of reaction time at (a) –0.4 V and (b) –0.6 V in 0.1 M KNO_3 + 0.1 M K_2SO_4, pH 11.5.

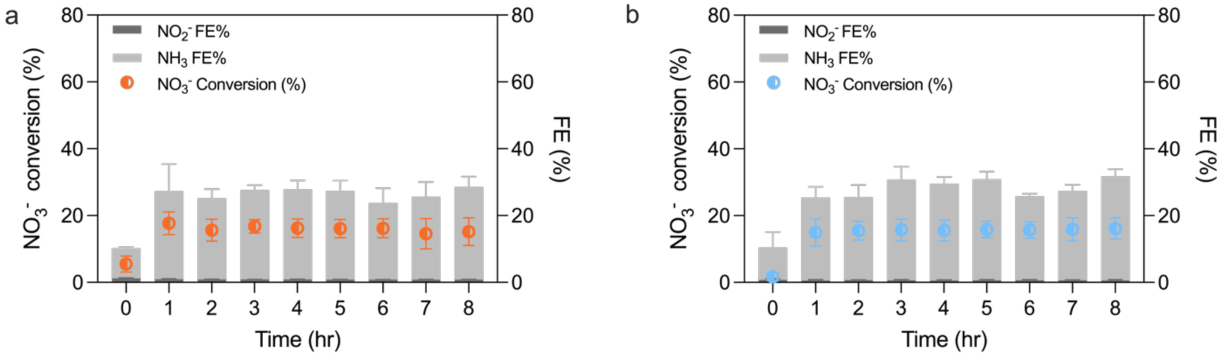

Figure S13. Nitrate conversion and Faradaic efficiency from flow-by experiments for (a) Cu_1 and (b) Cu_{NP} operated at −20 mA cm^{-2} with a flow rate of 0.1 mL min^{-1}. The Faradaic efficiency for nitrite is minimal. Conditions: 0.1 M KNO_3 + 0.1 M K_2SO_4, pH 11.5.

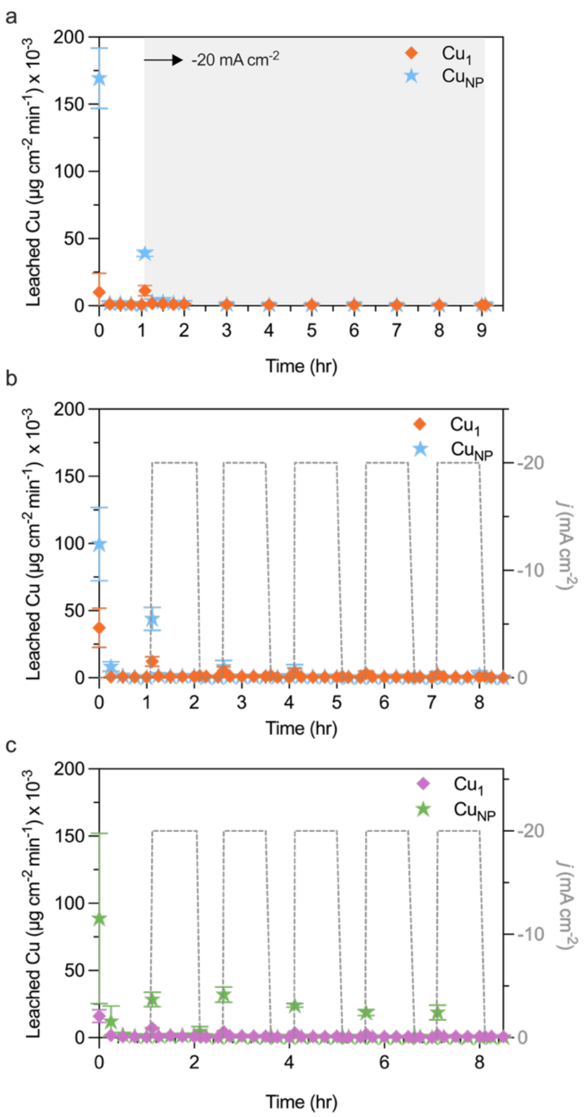

Figure S14. Copper leaching profiles during flow-by nitrate reduction and pulsing experiments. (a) Copper leaching profiles for Cu_1 and Cu_{NP} operated at −20 mA cm^{-2} for 8 h in 0.1 M KNO_3 + 0.1 M K_2SO_4,pH 11.5. Samples were taken every 15 minutes for 1 hour at OCV, every 15 minutes during the first hour of electrolysis followed by hourly sampling for the remainder of the reaction. A final sample was taken post-reaction at OCV. Catalyst pulsing experiments for Cu_1 and Cu_{NP} conducted in (b) nitrate (0.1 M KNO_3 + 0.1 M K_2SO_4, pH 11.5) and (c) HER conditions (0.15 M K_2SO_4, pH 11.5). Electrodes were equilibrated at OCV for 1 h prior to applying −20 mA·cm^{-2} for 1 h, followed by a 30 min OCV period. Copper leaching during both on and off periods was monitored, and the cycle was repeated five times.

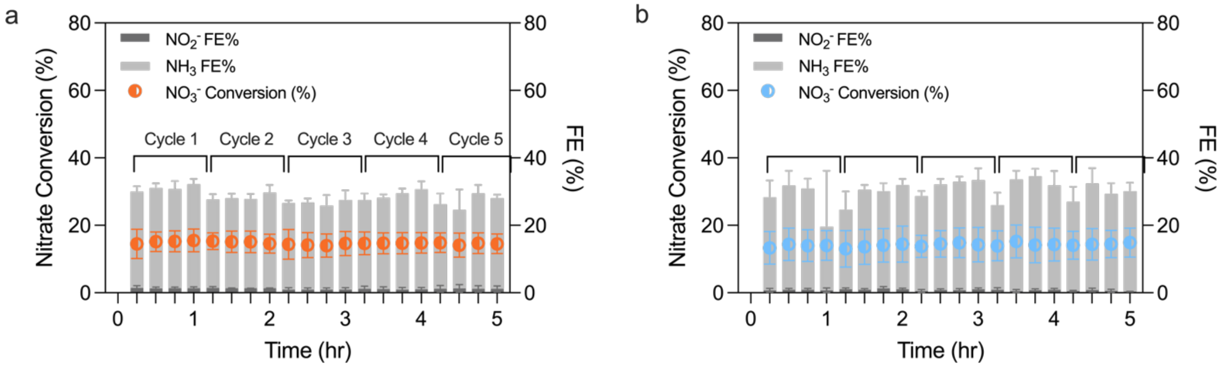

Figure S15. Nitrate conversion and Faradaic efficiency during flow-by "on/off" pulsed operation experiments for (a) Cu_1 and (b) Cu_{NP} at −20 mA cm^{-2} in 0.1 M KNO_3 + 0.1 M K_2SO_4, pH 11.5. The 15-minute "off" periods at OCV between each cycle are not shown. Individual cycles are indicated by brackets. Faradaic efficiency and nitrate conversion remain consistent across consecutive cycles.

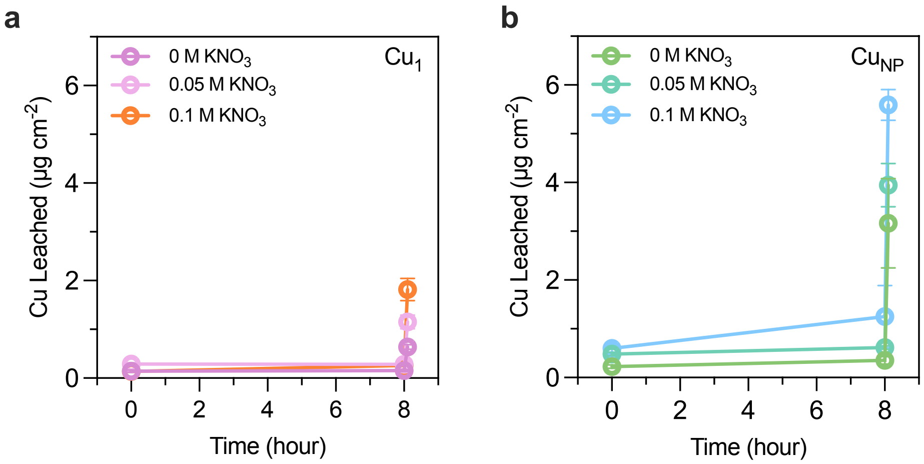

Figure S16. Copper leaching as a function of nitrate concentration. Copper leaching in electrolytes containing no added nitrate (0.15 M K_2SO_4, pH 11.5) and halved nitrate concentration (0.05 M KNO_3, pH 11.5), compared to 0.1 M KNO_3 condition, during 8-hour batch electrolysis at −0.8 V for (a) Cu_1 and (b) Cu_{NP}. Sampling procedure is outlined in **Figure 2a**.

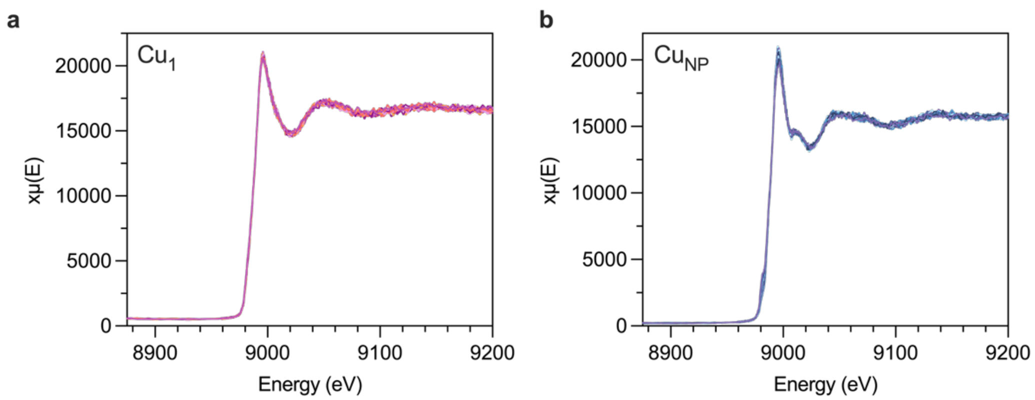

Figure S17. Unnormalized *in situ* XANES spectra of (a) Cu_1 and (b) Cu_{NP} collected during the OCV period prior to NO_3RR. No significant changes were observed during this ~30 min equilibrium period.

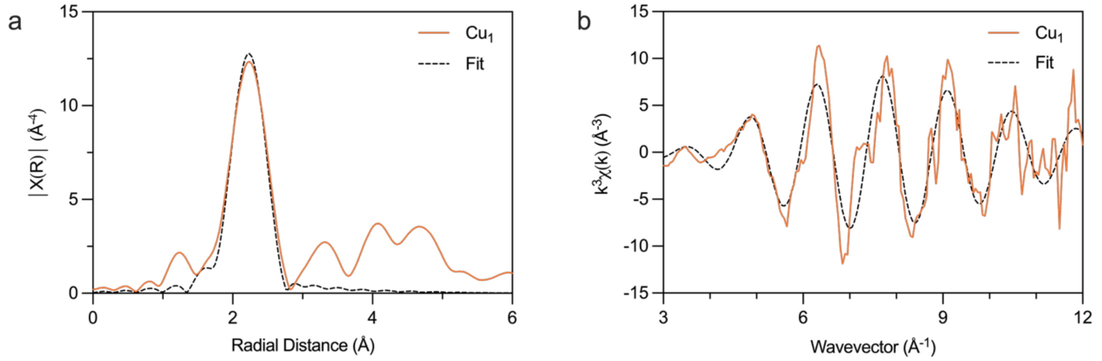

Figure S18. EXAFS fits of Cu_1 measured *in situ* at −0.8 V in 0.1 M KNO_3 + 0.1 M K_2SO_4, pH 11.5: (a) R-space and (b) k^3-space. R-space fit ranged from 1-3 Å and k-space fit ranged from 3-12 $Å^{-1}$.

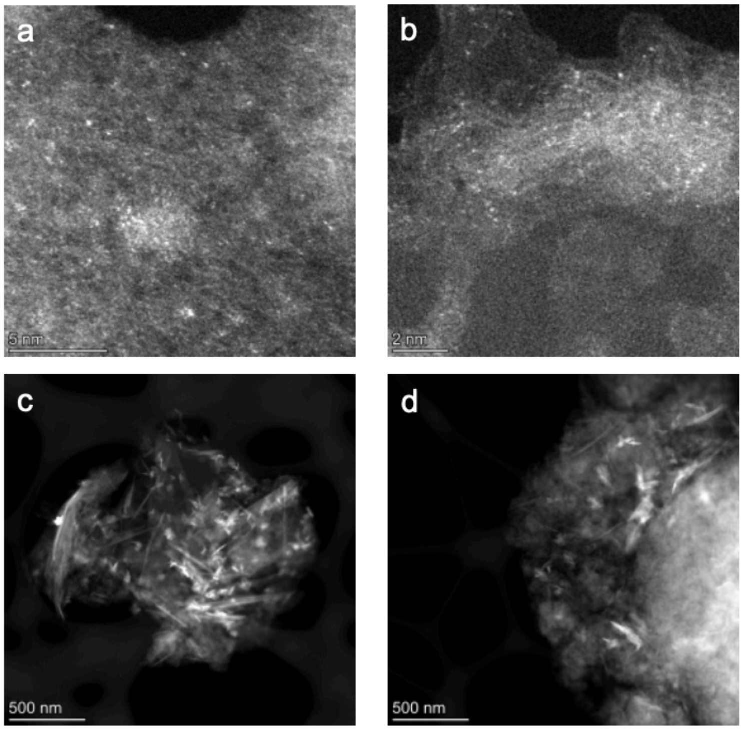

Figure S19. HAADF-STEM images of post-reaction electrodes (8 hours at −0.8 V in 0.1 M KNO_3 + 0.1 M K_2SO_4, pH 11.5) for (a,b) Cu_1 and (c,d) Cu_{NP}. After the reaction, the Cu_1 shows redistribution of single atoms into higher-density regions, while Cu_{NP} catalyst exhibits particle growth to ~ 200 nm with an oblong morphology.

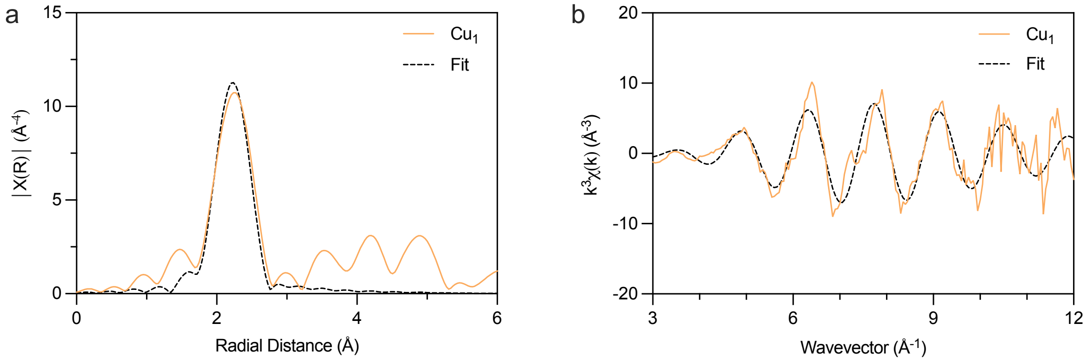

Figure S20. EXAFS fits of Cu_1 measured *in situ* at −0.8 V in 0.15 M K_2SO_4, pH 11.5: (a) R-space and (b) k^3-space. R-space fit ranged from 1-3 Å and k-space fit ranged from 3-12 $Å^{-1}$.

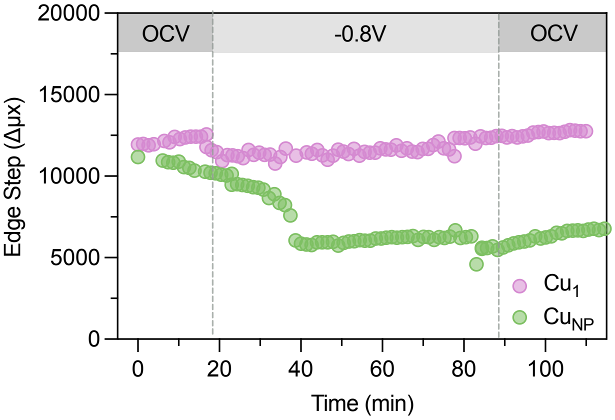

Figure S21. Time evolution of edge step during *in situ* XAS for Cu_1 and Cu_{NP} in the nitrate-free electrolyte (−0.8 V in 0.15 M K_2SO_4, pH 11.5).

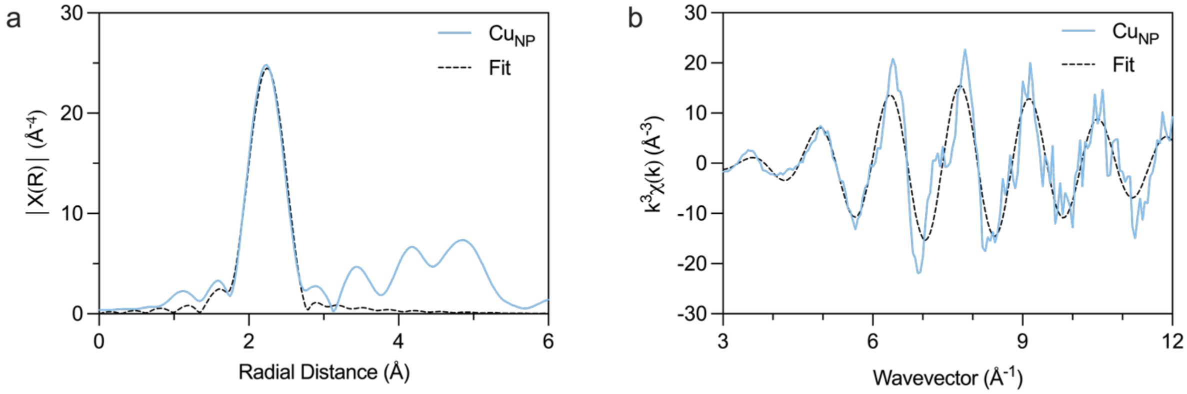

Figure S22. EXAFS fits of Cu_{NP} measured *in situ* at −0.8 V in 0.1 M KNO_3 + 0.1 M K_2SO_4, pH 11.5: (a) R-space and (b) k^3-space. R-space fit ranged from 1-3 Å and k-space fit ranged from 3-12 $Å^{-1}$.

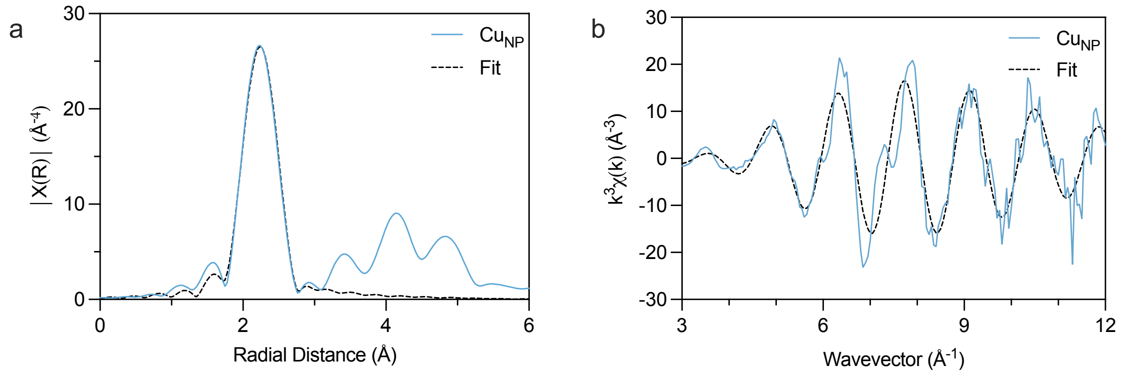

Figure S23. EXAFS fits of Cu_{NP} measured *in situ* at −0.8 V in 0.15 M K_2SO_4, pH 11.5: (a) R-space and (b) k^3-space. R-space fit ranged from 1-3 Å and k-space fit ranged from 3-12 Å^{-1}.

Sample	Scattering Path	CN	R (Å)	σ^2	ΔE0
Cu_1	Cu-N	4.10±0.25	1.954±0.002	0.0097±0.0024	−3.30±2.15
Cu_{NP}	Cu-Cu	5.98±0.17	2.548±0.012	0.0087±0.0013	4.98±1.65
Cu_{NP}	Cu-O	1.00±1.13	1.875±0.074	0.0035±0.0049	9.09±4.21

Table S1. Best-fit parameters extracted from FT-EXAFS of *ex situ* Cu_1 and Cu_{NP}. CN = coordination number, R = radial distance, σ^2 = Debye-Waller factor, and ΔE0 = edge energy shift.

Catalyst	Electrolyte	Potential (V vs RHE)	Duration (hr)	$Cu_{total}/Cu_{initial}$ (%)
Cu_1	0.1 M KNO_3 + 0.1 M K_2SO_4, pH 11.5	–0.4	1	7.8±0.9
			3	7.1±0.8
			5	10±2.3
			8	12±0.1
		–0.6	1	9.1±1.1
			3	10±1.5
			5	10.6±0.7
			8	13±1.8
		–0.8	1	9.4±0.3
			3	9.8±0.6
			5	11±3.0
			8	12±1.6
	0.05 M KNO_3 + 0.12 M K_2SO_4, pH 11.5	–0.8	8	8.0±1.0
	0.15 M K_2SO_4, pH 11.5	–0.8	8	4.5±0.3
Cu_{NP}	0.1 M KNO_3 + 0.1 M K_2SO_4, pH 11.5	–0.4	1	8.9±1.1
			3	14.8±1.9
			5	14.8±1.6
			8	13.7±2.1
		–0.6	1	12±0.7
			3	13±1.5
			5	17±3.0
			8	15±0.8
		–0.8	1	11±1.6
			3	15±1.3
			5	16±2.4
			8	18±1.0
	0.05 M KNO_3 + 0.12 M K_2SO_4, pH 11.5	–0.8	8	13±4.1
	0.15 M K_2SO_4, pH 11.5	–0.8	8	10±3.0

Table S2. Total Cu leaching as a percentage of initial Cu on the electrode surface for batch experiments.

Catalyst	Electrolyte	Current (mA cm^{-2})	Duration	$Cu_{sum}/Cu_{initial}$ (%)
Cu_1	0.1 M KNO_3 + 0.1 M K_2SO_4, pH 11.5	–20	8 h	1.2±0.4
	0.1 M KNO_3 + 0.1 M K_2SO_4, pH 11.5	–20	5, 1-h cycles	3.0±1.2
	0.15 M K_2SO_4, pH 11.5	–20	5, 1-h cycles	2.6±0.6
Cu_{NP}	0.1 M KNO_3 + 0.1 M K_2SO_4, pH 11.5	–20	8 h	1.6±0.3
	0.1 M KNO_3 + 0.1 M K_2SO_4, pH 11.5	–20	5, 1-h cycles	2.6±0.8
	0.15 M K_2SO_4, pH 11.5	–20	5, 1-h cycles	4.0±0.4

Table S3. Total Cu leaching due to NO_3RR as a percentage of initial Cu on the electrode surface for flow-by experiments. The 1-h equilibration period was excluded from leaching calculations.

Electrolyte	Conductivity (mS cm^{-1})
0.1 M KNO_3 + 0.1 M K_2SO_4, pH 11.5	29.58
0.05 M KNO_3 + 0.12 M K_2SO_4, pH 11.5	30.28
0.15 M K_2SO_4, pH 11.5	29.72

Table S4. Conductivity of electrolyte solutions.

Sample	Scattering Path	CN	R (Å)	σ^2	ΔE0	Electrolyte
Cu_1	Cu-Cu	6.63±0.16	2.540±0.021	0.0090±0.0019	3.13±2.12	0.1 M KNO_3 + 0.1 M K_2SO_4 (pH 11.5)
Cu_{NP}	Cu-Cu	11.60±0.09	2.539±0.021	0.0085±0.0008	5.48±0.94	0.1 M KNO_3 + 0.1 M K_2SO_4 (pH 11.5)
Cu_1	Cu-Cu	5.30±0.20	2.534±0.026	0.0084±0.0021	3.49±2.42	0.15 M K_2SO_4 (pH 11.5)
Cu_{NP}	Cu-Cu	10.94±0.09	2.539±0.022	0.0074±0.0005	4.47±0.63	0.15 M K_2SO_4 (pH 11.5)

Table S5. Best-fit parameters extracted from FT-EXAFS of *in situ* Cu_1 and Cu_{NP} at −0.8 V in their respective electrolytes. CN = coordination number, R = radial distance, σ^2 = Debye-Waller factor, and ΔE0 = edge energy shift. The coordination number attributed to the Cu-Cu scattering path increases to a greater degree during a negative applied potential in the presence of nitrate.
